# Global, regional, and national burden and temporal trends of depressive disorders in women of child-bearing age, 1990 to 2021: A worldwide analysis

**DOI:** 10.1097/MD.0000000000045215

**Published:** 2025-10-24

**Authors:** Tiansheng Zhu, Ying Fan, Ke Jin, Yunhan Shen, Xiayan Ye

**Affiliations:** a College of Mathematics and Computer Science, Zhejiang A & F University, Hangzhou, China; b Wenzhou People’s Hospital, Wenzhou Maternal and Child Health Care Hospital, The Wenzhou Third Clinical Institute Affiliated To Wenzhou Medical University, The Third Affiliated Hospital of Shanghai University, Wenzhou, Zhejiang, China.

**Keywords:** AAPC, depressive disorders, global burden of disease study, WCBA

## Abstract

As a severe public health issue, depressive disorders (DD) have caused an increasing burden of disease. The global status of women’s DD is underestimated, particularly the burden on women of child-bearing age (WCBA). We aim to investigate the pattern and trend of DD among WCBA from 1990 to 2021. We retrieved data from the Global Burden of Disease Study 2021 on the incidence, prevalence, and disability-adjusted life years (DALYs) in 204 countries and territories from 1990 to 2021. And we calculated the average annual percentage changes in age-standardized incidence rates, age-standardized prevalence rates, and DALYs rates, stratified by age and socio-demographic index (SDI), to quantify temporal trends. Additionally, a Bayesian age–period–cohort model was employed to project age-standardized rates up to 2030. Spearman correlation analysis was used to examine the correlation between age-standardized rates and SDI. In 2021, globally, the number of DALYs of DD among WCBA increased by 69% from 12.4 million to 21.0 million compared to 1990. The global burden of DD among WCBA has significantly increased from 1990 to 2021, with projections indicating continued growth. The average annual percentage changes from 1990 to 2021 for the age-standardized DALYs rates, age-standardized prevalence rates, and age-standardized incidence rates all demonstrated a highly significant positive correlation with SDI levels. Globally, the DALYs rate, incidence rate, and prevalence rate of DD increase with age. The rising global incidence of DD, coupled with regional variations in prevalence and DALYs, underscores the urgent need for innovative prevention and healthcare strategies to mitigate the burden among WCBA worldwide.

## 1. Introduction

Depressive disorders (DD), as a subset of mental disorders, are a common emotional disorder caused by factors such as neurotransmitter imbalance, genetics, psychological stress, inflammation, and nerve damage.^[1]^ Mental disorders remain among the leading causes of global burden, and a large proportion of the world’s disease burden is attributable to them.^[2]^ Research has revealed that mental disorders are a significant factor in suicidal behavior, with DD being the primary mental risk factor.^[3]^ Depression is recognized as the predominant contributor to the global burden of mental health-related diseases and serves as the leading cause of disability worldwide.^[4]^ Between 2010 and 2021, among the 25 leading level 3 causes, age-standardized disability-adjusted life year (DALY) rates increased most substantially for anxiety disorders (16.7% [14·0–19·8]), depressive disorders (DD) (16.4% [11·9–21·3]), and diabetes (14.0% [10·0–17·4]).^[5]^ As a major mental disorder, DD have attracted increasing attention from policymakers and health managers in related research.^[6]^ Predictive modeling indicates that by 2030 they will rank as either the second^[7]^ or the leading contributor to the global burden of disease (GBD).^[8]^

Studies have shown that DD events occur across all age groups but are predominantly found in the working age population (15–59 years).^[9]^ Other studies have shown that DD among the top 10 (6th) cause of DALYs in people aged 10 to 49 years.^[4,10]^ Across the lifespan, depression is almost twice as common in women as in men, and this disparity persists throughout the childbearing years.^[8,11]^ Globally, the average DALY for depression is higher in women than in men of the same age, and higher in women of childbearing age (WCBA, between 15 and 49 years) than in younger (<15 years) and older (≥50 years) women.^[12]^ Compared with the general population, WCBA exhibits heightened susceptibility to depression.^[13]^ Pregnancy is a salient trigger for DDs in WCBA and is linked to adverse outcomes such as low birth weight, maternal suicide, and preterm birth. The rising burden of depression in this group compromises individual well-being and reverberates through family dynamics and broader community health. These warrant further investigation into the burden of DD among this special population. Currently, most studies focus on children, adolescents, or the elderly,^[6,14–16]^ or on all age groups collectively.^[9,17]^ However, few have specifically quantified the burden of depression in WCBA, leaving a critical gap in understanding its impact on fertility and family health.

Socio-demographic index (SDI), which represents the level of economic development, is closely related to depressive symptoms.^[18]^ It provides a framework for understanding how socio-economic factors influence the prevalence and burden of mental disorders, including DD. Mendelian randomization studies employ genetic variants as instrumental variables to establish causal pathways from poverty to mental illness.^[19]^ These findings imply a causal link between depression onset or progression and SDI. Therefore, examining the relationship between SDI and depression among WCBA is essential.

To strengthen epidemiological evidence and guide intervention priorities, we conducted a comprehensive analysis of temporal trends in DD among WCBA from 1990 to 2021. Using Global Burden of Disease 2021 data, we examined prevalence, incidence, and DALYs across global, regional, and national strata, stratified by SDI levels. We also assessed determinants influencing DALYs in this population.

## 2. Methods

### 2.1. Data sources and data collection

In this investigation, we procured comprehensive data on the incidence, prevalence, and DALYs for DD among WCBA, stratified by geographical regions (21 GBD regions), locations (204 countries and territories), age groups (the age range of 15–49 years old was divided into 7 groups), and time periods (from 1990–2021). The detailed information and original data were obtained from the Global Health Data Exchange query tool (https://ghdx.healthdata.org/gbd-results-tool). The analysis encompassed both crude rates and age-standardized rates (ASR) to account for population structure variations as previously described.^[20]^ DisMod-MR 2.1, a Bayesian model-based meta-regression tool^[5]^ was employed to estimate the incidence, prevalence, and DALYs associated with depression across various regions and countries.

The disease categories of DD (ICD-10 F32-F33.9, F34.1) are divided into 2 major categories in GBD studies, including major depressive disorder (ICD-10 F32-F33.9) and dysthymia (ICD-10 F34.1).^[14,21,22]^ Nations across the globe are sorted into 5 distinct SDI brackets, which comprise low, low-middle, middle, high-middle, and high SDI regions, as per the SDI values reported by the institute for health metrics and evaluation in the GBD Study 2021. An elevated SDI typically signifies elevated per capita income, advanced educational attainment, diminished fertility rates, and enhanced healthcare provision in the region.^[5]^

Furthermore, in accordance with the World Health Organization’s criteria, the demographic subset of women considered to be within their reproductive years, denoted as WCBA, is specifically delineated by the age range of 15 to 49 years.^[23]^ Our analysis spanned 7 distinct age brackets (15–19, 20–24, 25–29, 30–34, 35–39, 40–44, and 45–49 years) for WCBA.

The calculation of DALYs incorporates both years of life lost due to premature mortality and years lived with disability, providing a comprehensive measure of disease burden that extends beyond mortality alone.^[24]^ The observed changes in DALYs over time offer valuable insights into the evolving health landscape and can inform public health strategies and resource allocation.

### 2.2. Statistical analysis

Data and their respective rates were extracted directly from the GBD Study 2021.^[25]^ All rate estimates are presented per 100,000 individuals, and the 95% uncertainty intervals (UIs) were calculated by the GBD by utilizing the 2.5th and 97.5th percentiles from a distribution of 1000 ordered estimates.^[26]^ A descriptive analysis was performed to characterize the burden of DD among WCBA on a global scale. We conducted a comparative analysis of the age-standardized prevalence, incidence, and DALYs (all per 100,000 population) among WCBA with DD across various age groups, genders, regions, and nations. Utilizing data on DD and related risk factors from the GBD Study, we computed the ASRs along with their respective 95% confidence intervals (CIs). These calculations were based on the world standard population as documented in the GBD Study 2021, allowing for regional comparisons. Furthermore, we employed Joinpoint regression method^[27]^ to determine the average annual percentage change (AAPCs), which served as a metric for assessing the temporal trends. The results are presented per 100,000 population, employing the formula in Eq. (1). The AAPCs indicate the average annual increase or decrease in a specific metric over a defined time span. A positive (or negative) AAPC value signifies an upward (or downward) trend.^[27]^ The AAPC was derived using the formula in Eq. (2). ASRs per 100,000 people of WCBA were calculated according to the formula (1)^[4]^:

$$\begin{array}{l} ASR=\frac{\overset{N}{\underset{i=1}{\sum }}(age_{i}\times weight_{i})}{\overset{N}{\underset{i=1}{\sum }}weight_{i}} \end{array}$$ (1)

In the equation, $age_{i}$ is the age-specific rate in the *i*^th^ age class and $weight_{i}$ is the weight in the same age subclass of the chosen reference standard population. N is the upper age upper bound.

$$\begin{array}{l} AAPC=\left\{exp\left(\frac{\sum weight_{i}\beta_{i}}{\sum weight_{i}}\right)-1\right\}\times 100 \end{array}$$ (2)

Where the “exp” function refers to the exponential function with natural logarithm base e, $weight_{i}$ is the length of $i^{th}$ segment in the range of years, and $\beta_{i}$ is the corresponding slope coefficient.

We conducted an analysis to determine the correlation between the SDI and the ASRs of incidence, prevalence, and DALYs for WCBA with DD as in literature.^[28]^ This analysis utilized locally weighted regression to assess the relationship. Furthermore, Spearman rank correlation tests were applied to quantify these correlations. A statistically significant association was inferred at the *P* < .05 level, while a highly significant association was denoted by *P* < .001, indicating the strength of the relationship between the variables under study.

The Bayesian age–period–cohort (BAPC) model was utilized to predict the burden of DD from 2021 to 2030, employing the methodology described in previously published paper.^[20,29,30]^ The computational analysis was conducted utilizing the R-package BAPC, adhering to methodological frameworks validated in precedent literature.^[31]^

Statistical analytical procedures were conducted using joinpoint regression program (version 5.2.0), and R statistical language (version 4.4.1).

## 3. Results

### 3.1. Global trends of overall DD among WCBA

Globally, the number of DALYs of DD among WCBA increased by 69% between 1990 and 2021, from 12.4 million to 21.0 million, and the global age-standardized rate of DALY increased by 13.1% between 1990 and 2021, from 948.9 (95% UI: 629.49–1341.1) to 1073.5 (95% UI: 707.59–1515.33) per 100,000, with an AAPC of 0.45% (Table 1). The global prevalence among WCBA people increased by 67.6% between 1990 and 2021, from 72.3 million to 121.2 million, and the global age-standardized prevalence rate (ASPR) increased by 11.3%, from 5545.28 to 6173.45 per 100,000, with an AAPC of 0.38% (Table S1, Supplemental Digital Content, https://links.lww.com/MD/Q399). Incidence was similar to that of prevalence, with a larger AAPC of 0.53% (Table S2, Supplemental Digital Content, https://links.lww.com/MD/Q399).

**Table 1:** The number, age-standardized rate of DALYs for depressive disorders among WCBA in 1990 and 2021, and their temporal trends from 1990 to 2021.

Variables	DALYs in 1990 (95% UI)		DALYs in 2021 (95% UI)		AAPC (95% CI)
	Number (no. ×10^3^) (95% UI)	Age-standardized rate per 100,000 (95% UI)	Number (no. ×10^3^) (95% UI)	Age-standardized rate per 100,000 (95% UI)	For rate (95% CI)
Global	12445.34 (8084.22–18023.44)	948.86 (629.49–1341.1)	21042.42 (13468.19–30593.48)	1073.5 (707.59 - 1515.33)	0.45 (0.33–0.57)
SDI					
High SDI	2291.81 (1525.89–3251.7)	1007.25 (687.24–1396.84)	3350.6 (2211.22–4819.02)	1399.31 (948.19–1966.38)	1.2 (1.13–1.26)
High-middle SDI	2449.32 (1601.46–3518.04)	889.89 (603.11–1230.84)	2939.22 (1878.57–4277)	939.53 (620.9–1320.53)	0.19 (0.03–0.36)
Middle SDI	3677.23 (2385.35–5345.05)	850.96 (571.44–1195.06)	5819.96 (3723.68–8429.63)	926.08 (615.52–1294.2)	0.34 (0.2–0.47)
Low-middle SDI	2832.46 (1799.43–4175.68)	1088.11 (716.74–1543.47)	5817.17 (3674.56–8529.56)	1164.37 (776.76–1616.82)	0.24 (0.01–0.47)
Low SDI	1183.84 (747.04–1751.94)	1116.33 (726.17–1599.62)	3099.94 (1938.03–4603.05)	1179.58 (774.68–1664.36)	0.16 (-0.1–0.42)
Subtypes					
MDD	10153.27 (6357.29–15247.17)	770.16 (492.41–1129.11)	17434.39 (10680.48–26207.97)	891.02 (562.21–1299.98)	0.54 (0.41–0.68)
Dysthymia	2292.07 (1378.01–3426.14)	178.69 (109.69–261.29)	3608.03 (2170.2–5433.4)	182.48 (113.01–266.7)	0.06 (0.01–0.10)
Region					
Andean Latin America	69.13 (42.31–105.17)	751.79 (488.78–1083.79)	169.29 (101.54–261.07)	966.63 (623.61–1402.43)	0.95 (0.45–1.45)
Australasia	73.94 (48.09–106.57)	1380.17 (956.85–1872.55)	110.9 (68.36–167.97)	1563.09 (1021.41–2243.42)	0.46 (0.41–0.51)
Caribbean	106.49 (66.72–161.46)	1161.78 (769.85–1669.22)	144.58 (87.04–222.58)	1195.95 (772.38–1714.36)	0.16 (0.1–0.21)
Central Asia	125.7 (79.39–185.29)	772.01 (504.65–1098.3)	220.83 (137.42–332.91)	903.45 (628.22–1233.93)	0.6 (0.51–0.68)
Central Europe	215.91 (137.3–313.41)	695.87 (456.47–979.85)	211.07 (132.81–308.76)	788.38 (516.55–1114.17)	0.46 (0.37–0.54)
Central Latin America	316.37 (199.59–471.54)	791.2 (519.52–1126.5)	800.33 (496.09–1189.26)	1168.22 (756.74–1658.62)	1.51 (1.25–1.78)
Central Sub-Saharan Africa	190.21 (117.32–291.34)	1597.25 (1070.6–2254.06)	535.68 (313.87–827.73)	1695.2 (1231.78–2162.85)	0.27 (0.08–0.47)
East Asia	2490.86 (1624.45–3611.96)	766.37 (536.27–1033.19)	2009.85 (1320.29–2848.27)	560.51 (393.16–745.34)	-0.96 (-1.24–0.68)
Eastern Europe	486.39 (309.31–712.82)	864.27 (583.21–1200.35)	546.45 (346.41–807.15)	1083.11 (766.31–1458.89)	0.73 (0.62–0.84)
Eastern Sub-Saharan Africa	481.18 (303.36–708.08)	1188.39 (774.63–1681.48)	1302.6 (802.6–1945.05)	1273.15 (827.63–1802.56)	0.26 (0.2–0.31)
High-income Asia Pacific	280.95 (183.63–402.27)	618.37 (425.19–842.32)	290.04 (188.98–417.63)	783.79 (536.54–1073.71)	0.81 (0.7–0.93)
High-income North America	886.75 (586.93–1271.51)	1193.29 (840.3–1616.26)	1597.17 (1063.93–2290.9)	1929.21 (1374.01–2605.45)	1.64 (1.36–1.92)
North Africa and Middle East	1005.85 (626.77–1502.45)	1323.9 (851.25–1913.98)	2420.07 (1465.3–3673.29)	1515.15 (953.16–2216.53)	0.57 (0.3–0.83)
Oceania	10.69 (6.68–16.23)	698.73 (477.68–974.96)	24.89 (14.83–38.3)	720.59 (479.2–1008.14)	0.08 (0.01–0.15)
South Asia	2645.82 (1692.29–3895.33)	1088.15 (741.79–1503.86)	5462.96 (3482.82–7949.8)	1118.19 (789.72–1471.64)	0.05 (-0.33–0.43)
Southeast Asia	692 (446.21–1012.84)	593.89 (396.31–837.31)	1260.59 (802.92–1855.94)	683.77 (453.27–967.29)	0.46 (0.44–0.48)
Southern Latin America	132.1 (85.58–194.5)	1063.1 (725.69–1486.47)	210.97 (131.45–313.05)	1221.46 (839.46–1653.25)	0.56 (0.21–0.91)
Southern Sub-Saharan Africa	135.54 (88.23–197.11)	1080.34 (762.42–1446.14)	288.12 (185.61–421.68)	1329.3 (944.35–1769.01)	0.81 (0.59–1.03)
Tropical Latin America	477.48 (309–697.27)	1225.46 (852.8–1667.89)	876.6 (555.83–1277.43)	1419.51 (1044.52–1827.17)	0.58 (0.53–0.63)
Western Europe	1207.94 (802.91–1707.06)	1258.72 (853.18–1749.01)	1429.19 (913.49–2114.35)	1527.48 (1003.48–2204.91)	0.71 (0.45–0.97)
Western Sub-Saharan Africa	414.03 (258.81–611.94)	1017.39 (664.51–1441.16)	1130.24 (707.47–1676.79)	996.09 (664.34–1386.86)	-0.03 (-0.21–0.14)

AAPC = average annual percentage change, CI = confidential interval, DALYs = disability-adjusted life years, DD = depressive disorder, MDD = major depressive disorder, No = number, SDI = socio-demographic index, UI = uncertainty interval, WCBA = women of childbearing age.

The global temporal trends of overall DD among WCBA from 1990 to 2021 exhibit significant growth. The age-standardized DALYs rate, ASPR, and age-standardized incidence rate (ASIR) all exhibited relative stability between 1990 and 2005. Over the subsequent 5 years, these rates declined annually. Following this period, a gradual upward trend was observed. From 2019 to 2020, there was a sharp increase, after which a slow rise continued through to 2021 (Figure S1, Supplemental Digital Content, https://links.lww.com/MD/Q398).

### 3.2. Global trends by socio-demographic index

In 2021, among the 5 SDI regions, the highest age-standardized DALYs rate for DD was observed in high SDI regions, with a value of 1399.31 (per 100,000), followed by the low SDI regions (1179.58 per 100,000) while the lowest value was found in middle SDI regions, with a value of 926.08 (per 100,000) (Table 1). Similarly, high SDI regions had the highest ASPR and ASIR, whereas middle SDI regions had the lowest for both metrics (Table 1). The temporal trends of DALYs for DD among WCBA observed from 1990 to 2021 across all 5 SDI regions indicate an increase, with the most significant rise noted in regions characterized by a high SDI, where the AAPC reached 1.2% (Table 1). In contrast, the increase in other regions was comparatively modest, with an AAPC of 0.34% or lower (Table 1). The trends in ASPR and ASIR demonstrate a consistent pattern (Tables S1 and S2, Supplemental Digital Content, https://links.lww.com/MD/Q399). The DALY, prevalence, and incidence cases for the 5 SDI regions in the years 1990 and 2021 are delineated in Table 1, Tables S1 and S2, Supplemental Digital Content, https://links.lww.com/MD/Q399, with corresponding ASRs aligning with the data. Then, line charts were generated for the annual age-standardized DALY rate, ASPR, and ASIR across these SDI regions and global region (Figure S1, Supplemental Digital Content, https://links.lww.com/MD/Q398). From the charts, it can be observed that these patterns exhibit unique characteristics. For the age-standardized DALYs rate, high SDI region, low SDI region, and low-middle region have exhibited a trend characterized by an initial gradual increase, subsequent decline, and subsequent ascent. Conversely, the middle SDI region and the high-middle SDI region initially experienced a gradual decline, followed by a slow increase, and culminating in a rapid ascent (Figure S1A, Supplemental Digital Content, https://links.lww.com/MD/Q398). Overall, DALY, incidence, and prevalence trends were consistent across the 5 SDI regions (Figure S1A–C, Supplemental Digital Content, https://links.lww.com/MD/Q398).

### 3.3. The association between ASR, AAPC, and SDI

**Figure 1.**
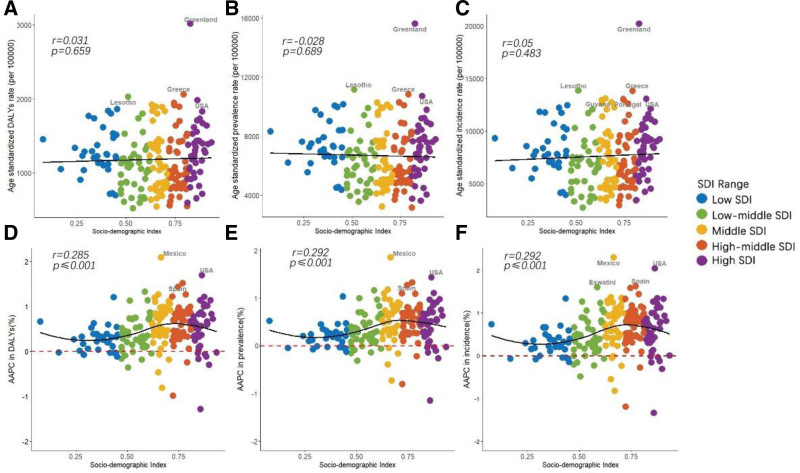
DD related burden and their AAPCs with different SDI levels. Age-standardized DALYs (A), prevalence (B), and incidence (C) rates of WCBA with DD in 2021, respectively, as well as the average annual percentage changes (AAPCs) in DALYs (D), prevalence (E), and incidence (F) from 1990 to 2021, are associated with the SDI. AAPC > 0 represents an increase in the rate, and AAPC < 0 represents a decrease in the rate. AAPC = average annual percentage change, DALYs = disability-adjusted life years, SDI = socio-demographic index, WCBA = women of childbearing age.

In 2021, the age-standardized DALYs rates, ASPR, and ASIR for WCBA with DD exhibited no significant correlation with SDI levels, with correlation coefficients (*r*) of 0.031 (*P* = .659) (Fig. 1A), -0.028 (*P* = .689) (Fig. 1B), and 0.05 (*P* = .483) (Fig. 1C), respectively. Greenland’s ASRs significantly exceed those of other nations for all 3 metrics, followed by Greece, Lesotho, and the USA. Guyana and Portugal likewise showed elevated ASIR. The age-standardized trend lines for these 3 metrics appear as nearly horizontal straight lines across these countries. However, the AAPC from 1990 to 2021 for these 3 metrics all demonstrated a highly significant positive correlation with SDI levels, with correlation coefficients (*r*) of 0.285 (Fig. 1D), 0.292 (Fig. 1E), and 0.292 (Fig. 1F), respectively, and *P*-values <.001 for each. Mexico, the USA, and Spain exhibited higher AAPC in age-standardized DALYs, ASIR, and ASPR compared with other countries. Eswatini also shows a notably higher AAPC in incidence rates. All 3 metrics of AAPC exhibit an S-shaped curve, increasing from low SDI to lower-middle and middle SDI regions, and then decreasing towards high-middle and high SDI areas. This pattern suggests a non-linear relationship between SDI and the burden of disease, indicating that the impact of socio-economic development on health outcomes may vary across different levels of development.

### 3.4. Global trends of regional disparities

**Figure 2.**
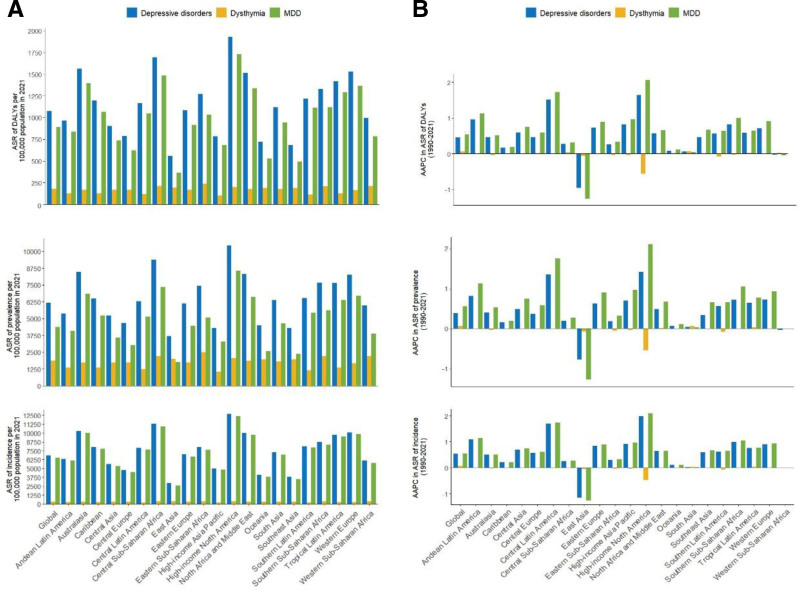
The association between DD subtypes and GBD regions. (A) Age-standardized incidence, prevalence and DALY rates in 2021, and (B) their AAPC from 1990 to 2021 for WCBA, globally and by 21 GBD regions. AAPC = average annual percentage change, DALYs = disability-adjusted life years, DD = depressive disorders, GBD = global burden of disease, WCBA = women of childbearing age.

Regional disparities still exist in 2021. Throughout the 21 GBD regions, the top 3 highest age-standardized DALY rates were found in high-income North America (1929.21 per 100,000), Central Sub-Saharan Africa (1695.2 per 100,000), and Australasia (1563.09 per 100,000) (Table 1, Fig. 2A). In contrast, the lowest rates were observed in East Asia (560.51 per 100,000), Southeast Asia (683.77 per 100,000), and Oceania (720.59 per 100,000) in 2021. High-income North America, Central Sub-Saharan Africa, and Australasia also ranked in the top 3 for ASIR and ASPR (Tables S1 and S2, Supplemental Digital Content, https://links.lww.com/MD/Q399, Fig. 2A). From 1990 to 2021, most of the 21 GBD regions exhibited increases in DALYs of DD among WCBA people (Table 1, Fig. 2B). The 2 regions with the highest average annual growth rates are high-income North America and Central Latin America, with AAPC reaching 1.64% and 1.51%, respectively. The region with the most significant negative growth is East Asia, with an AAPC of −0.96%.

### 3.5. Global trends of national disparities

**Figure 3.**
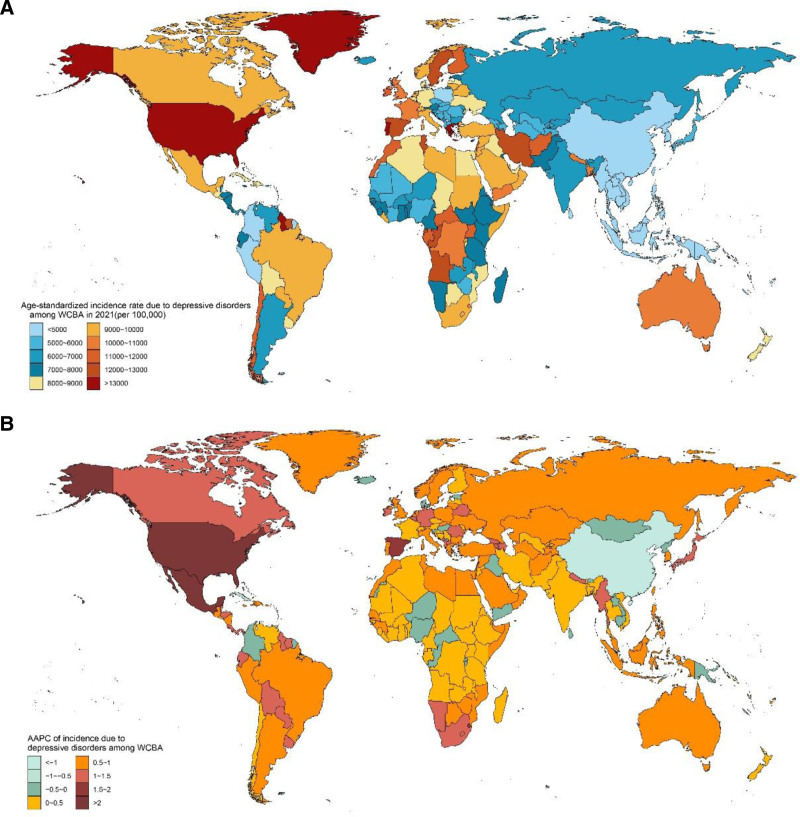
The spatial distribution of the age-standardized rate of incidence due to depressive disorders among WCBA in 204 countries and territories. (A) The rate of incidence due to depressive disorders among WCBA in 2021, (B) the AAPC of the rate of incidence due to depressive disorders among WCBA from 1990 to 2021. AAPC = average annual percentage change, WCBA = women of childbearing age.

Substantial variations in the incidence, prevalence, and DALYs of DD among WCBA are observed across nations (Fig. 3, Figures S2 and S3, Supplemental Digital Content, https://links.lww.com/MD/Q398). In 2021, Greenland recorded the highest ASIR (20,221 per 100,000), ASPR (15,623 per 100,000), and age-standardized DALYs rate (3016 per 100,000), whereas Myanmar had the lowest ASIR (2717) and DALYs (525), and Brunei Darussalam had the lowest ASPR (3156) (Fig. 3A and Tables S3–S5, Supplemental Digital Content, https://links.lww.com/MD/Q399). From 1990 to 2021, the ASIR increased in most of countries and regions (Fig. 3B). In more detail, a total of 177 countries and regions experienced positive growth, with Lebanon, Eswatini, Spain, the United States of America, and Mexico recording an AAPC above 1.5% (Fig. 3B and Table S6, Supplemental Digital Content, https://links.lww.com/MD/Q399). Conversely, 27 countries witnessed a decline in growth, and among them, Singapore and China experienced greatest reductions, with an AAPC below −1% (Table S6, Supplemental Digital Content, https://links.lww.com/MD/Q399).

### 3.6. Global trends by age subgroup

**Figure 4.**
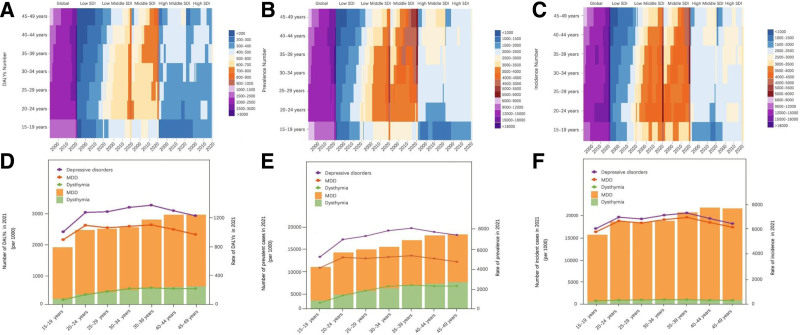
The longitudinal trends (1990–2021) and cross-sectional (2021) of DALYs, prevalence and incidence of WCBA with DD. Temporal changes in age distribution of (A) DALYs numbers, (B) prevalence numbers, and (C) incidence numbers of WCBA from 1992 to 2021 across SDI quintiles. Numbers and rates of DALYs, (D) prevalence cases (E), and incident cases (F). AAPC = average annual percentage change, DALYs = disability-adjusted life years, DD = depressive disorders, GBD = global burden of disease, SDI = socio-demographic index, WCBA = women of childbearing age.

The participants were divided into 7 age groups (15–19, 20–24, 25–29, 30–34, 35–39, 40–44, 45–49 years) to examine the age group distribution of DD and to calculate the proportion in each group. Figure 4A to C illustrated the variations in DALYs, prevalence, and incidence of DD across age groups among WCBA, stratified by SDI. Variations in DALYs, prevalence, and incidence across age groups and the 5 SDI regions were modest. Notably, the low-middle SDI and middle SDI regions exhibited relatively higher numbers, with the 15 to 19 group showing the smallest counts in these regions, while the high SDI and low SDI regions had relatively lower numbers. The age distribution of numbers and rates in DALYs and prevalence were largely consistent for the 2 subtypes and overall WCBA with DD globally (Fig. 4D and E). In 2021, both the DALYs and prevalence rates for major depressive disorder (MDD) and dysthymia increased steadily with age, reaching their highest in the 40 to 44 and 45 to 49 years age groups respectively (Fig. 4D and E). The age distribution of incidence numbers and rates for dysthymia exhibited a relatively stable pattern, while the incidence rate of MDD demonstrated a nearly gradual increase with advancing age (Fig. 4F). These patterns indicated limited age specificity in DD subtypes.. The numbers and rates of MDD were more than twice those of dysthymia across all 3 metrics. Especially in terms of incidence rates, MDD is significantly higher than dysthymia, with MDD rates being more than double those of dysthymia across all age groups in 2021.

### 3.7. The future trends predicted by BAPC

To predict future trends in DD, a BAPC analysis was conducted, providing prospective predictions of age-standardized DALYs rates, prevalence rates, and incidence rates up to 2030. Overall, the burden of DD is expected to continue to increase in the future (see Figure S4, Supplemental Digital Content, https://links.lww.com/MD/Q398). The projected ASRs of DALYs, prevalence, and incidence for WCBA suffering from DD in 2030 are estimated to be 1339.72 (Figure S4A, Supplemental Digital Content, https://links.lww.com/MD/Q398), 7829.73 (Figure S4B, Supplemental Digital Content, https://links.lww.com/MD/Q398), and 8232.29 (Figure S4C, Supplemental Digital Content, https://links.lww.com/MD/Q398) per 100,000 population, respectively.

## 4. Discussion

From 1990 to 2021, prevalence, incidence, and DALYs of DD among WCBA increased significantly. WCBA represents a distinct and vulnerable group for depression, characterized by the certain features, including biological, psychological, and socio-economic factors. Biologically, they face reproductive health concerns and hormonal fluctuations^[32]^ across the menstrual cycle, pregnancy, and postpartum periods that influence mood.^[33,34]^ Socially, they often balance multiple roles (including those of a daughter, wife, mother, and worker) and face economic challenges, increasing stress and depression risk.^[35]^ Economically, factors like gender pay gaps and limited opportunities exacerbate financial insecurity, contributing to DD.^[36]^ According to the GBD study, the burden of DD among WCBA exceeds that of the general population (Table 1, Tables S1 and S2, Supplemental Digital Content, https://links.lww.com/MD/Q399). The global age-standardized DALYs rate and prevalence rate for WCBA suffering from DD fall between those of young people (aged 10–24 years)^[14]^ and older adults (aged 60 years and above).^[6]^ Between 1990 and 2021, the global ASIR and ASPR of MDD in older adults increased annually by 0.24% (95% CI: 0.18–0.31) and 0.25% (95% CI: 0.19–0.30),^[37]^ respectively, compared to increases of 0.55% (95% CI: 0.40–0.70) and 0.55% (95% CI: 0.41–0.69) in WCBA (Tables S1 and S2, Supplemental Digital Content, https://links.lww.com/MD/Q399). The high burden of DD and rising trend of MDD among WCBA warrant sufficient attention from public health professionals. Projections indicate that the ASR of DD among WCBA will continue to rise steadily through 2030, posing significant challenges to global mental health systems. This population-specific increase in depression is anticipated to exacerbate existing disparities in healthcare access, strain existing mental health service delivery frameworks, and amplify the societal burden associated with untreated mental health conditions. The trajectory underscores the urgent need for integrated population-specific interventions that address both biological determinants and psychosocial stressors affecting women during their reproductive years.

Between 1990 and 2021, there were shifts in the regional balance of disease burden. In 1990, the regions with the highest disease burden rates were Central Sub-Saharan Africa, Australasia, and North Africa and Middle East, which aligns with previous research.^[16]^ Conversely, the regions with the lowest rates were the Pacific, Southeast Asia, and Central Europe. By 2021, the regions with the highest disease burden rates had shifted to High-income North America, Central Sub-Saharan Africa, and Australasia, while the lowest rates were observed in East Asia, Southeast Asia, and Oceania (Table 1). The ASIR and ASPR also exhibited shifts (Tables S1 and S2, Supplemental Digital Content, https://links.lww.com/MD/Q399). Across regional levels, the most rapid increases in the AAPC of age-standardized DALYs rates were observed in high-income North America with an AAPC of 1.64%, while East Asia experienced the most significant decline with an AAPC of −0.96% (Table 1, Fig. 2B). This aligns with the global trend of DD burden, where developed regions experience faster growth, while developing regions exhibit slower increases. We urge policymakers and stakeholders in regions with high disease burdens to develop action plans that prioritize interventions, allocate resources more effectively, and assess the impact of health policies and programs.

Our study revealed a multifaceted association between the SDI and depression, as illustrated in Figure S1, Supplemental Digital Content, https://links.lww.com/MD/Q398. Notably, the age-standardized DALYs rate, ASPR, and ASIR were markedly higher in high SDI regions in 2021, contrasting with the low SDI regions in 1990. This suggests a shift in the distribution of depression burden over time, aligning with the evolving SDI landscape. The incidence rate, prevalence rate, and DALYs rate of depression did not show a statistically significant correlation with SDI (Fig. 1A–C). Previous studies have also demonstrated an absence of significant correlation between the burden of mental disorders and the SDI.^[38]^ However, the AAPC of these metrics demonstrated a highly significant positive correlation with SDI regions (Fig. 1D–F). This suggests that while the immediate rates of depression do not directly correlate with economic development levels, the trends over time do exhibit a strong association, highlighting the dynamic nature of depression’s burden in relation to socio-economic progress. Previous research has established a significant positive correlation between a country’s per capita GDP and the risk of mood disorders, and it has been observed that areas with high SDI tend to have an increased risk of DD due to greater competitive pressure.^[39]^ Another factor is that socio-economic development in these regions has expanded mental health screening services, reduced stigma around mental illness, and consequently increased the reported prevalence of mental disorders.^[17]^ However, high SDI regions possess enhanced healthcare resources and social support, which are crucial in mitigating the burden of DD.^[40]^ This suggests that economic development and societal competitiveness may play a role in the incidence of mood disorders, particularly within the context of DD.^[18,39]^ Mental illness is more prevalent and persistent among lower socio-economic groups, and these individuals receive less treatment. Global epidemiological research has consistently shown a correlation between mental health conditions and socio-economic status.^[19,41]^ By leveraging the genetic information from genome-wide association studies, researchers can investigate the potential causal effects of socio-economic status on mental health, accounting for the influence of genetic factors and reducing the likelihood of confounding by reverse causation or unmeasured environmental variables.^[19]^ Low income is positively associated with attention deficit hyperactivity disorder, bipolar disorder, MDD, post-traumatic stress disorder, and schizophrenia, whereas moderate income exerts a protective effect on autism spectrum disorder and anorexia nervosa. High income, on the other hand, is positively linked to anorexia nervosa and autism spectrum disorder. This explains why the disease burden is highest in both high SDI and low SDI regions among the 5 SDI quintiles (Table 1 and Figure S1, Supplemental Digital Content, https://links.lww.com/MD/Q398). These findings indicate a complex bidirectional causal relationship between poverty and mental illness, with a potential for a vicious cycle.^[19]^

The COVID-19 pandemic reoriented global health priorities in 2020 toward controlling SARS-CoV-2 transmission and addressing surging healthcare demands. Since then, COVID-19 has transitioned from an emergent threat requiring acute response to an endemic infectious disease that populations must learn to live with and manage.^[5]^ Between 1990 and 2021, global trends in the ASRs of DALYs, ASIR, and ASPR for WCBA among DD followed a consistent trajectory (Figure S1, Supplemental Digital Content, https://links.lww.com/MD/Q398). From 2020 to 2021, these metrics rose sharply, coinciding with COVID-19-related restrictions on mobility and interpersonal contact that have been associated with elevated depression risk.^[42]^ The COVID-19 pandemic was associated with significant increases in the prevalence of anxiety and DDs, with women experiencing disproportionately greater mental health impacts.^[43]^ Evidence suggested that women’s psychological well-being is more sensitive to pandemic-related social and economic disruptions, partly due to heightened exposure to caregiving responsibilities, income loss, and domestic stressors.^[43,44]^ Actionable measures could center on strategies that are context-adapted, explicitly reach vulnerable populations, and are grounded in core public health principles: equity, stigma reduction, inclusivity, and the protection of human rights.^[45]^ Integrated service models (combining digital platforms, telehealth, and in-person care) could be flexibly adapted to meet individual needs. Public health policies can promote protective behaviors (regular sleep, exercise, balanced nutrition, and sustained social contact) and encourage timely professional help for persistent mood disorders.

The age effects displayed analogous patterns across various SDI regions, with a gradual and incremental increase as age increase (Fig. 4A–C). Our study indicates that the increasing trend of WCBA suffering from DD is predominantly concentrated within the elderly population, specifically among those aged 45 to 49 years. Aging itself is a major risk factor for depression, and women going through the menopausal transition may face an increased risk due to hormonal fluctuations. In terms of the impact of age on the prevalence, incidence, and DALYs of DD among WCBA, it appears that the influence of age is considerably less significant compared to the impact of the SDI. The SDI, which reflects a region’s socio-economic development, has a more profound effect on the burden of DD within this demographic. This suggests that while age is a factor, the socio-economic context plays a more dominant role in shaping the trends and patterns observed in DDs among WCBA. It is evident that for the subtypes of DD, the number of cases and rates of MDD are significantly higher than those of dysthymia (Fig. 4D–F). This may be attributed to the incomplete statistical data for dysthymia, as many individuals with dysthymia may not be aware of their condition and thus do not seek a diagnosis. Additionally, from the same figure, it is observed that both the number and rates of incidence exceed those of prevalence, suggesting that treatments for DD have been somewhat effective.

In this study, we utilized the BAPC model to investigate the future burden of DD among WCBA. We found a continued increase in DD cases until 2030. Compared to other models, the BAPC model is capable of more accurately predicting and reflecting the future burden of diseases in GBD research.^[20,46]^ The BAPC approach allows us to anticipate how the burden of depression may evolve over time, providing valuable insights for policymakers and public health practitioners. The standardized prevalence and incidence rates of depression among WCBA are much higher than the standardized rate of DALYs (Figures S1 and S4, Supplemental Digital Content, https://links.lww.com/MD/Q398), reflecting the severity of depression in this population and its significant impact on public health. To reduce the incidence and prevalence of depression among WCBA, comprehensive measures need to be taken, including strengthening mental health education, providing psychological support, and improving the social environment.

Given the substantial absolute and relative increases in the burden of mental disorders over the past 3 decades, it is imperative to enhance mental health services on both national and regional scales. This should involve prioritizing support for countries with the greatest needs. Comprehensive strategies should encompass primary care, specialized care, community-based services, and rehabilitative programs.^[22]^ The article could benefit from further investigation into the effectiveness of existing public health interventions and the prevalence of corrective measures, to provide more targeted recommendations for policy development.

## 5. Limitations

This research encounters certain constraints. First of all, in our work, we have employed GBD data, acknowledging their inherent limitations. Notably, data derived from the GBD model may contain errors due to flaws in the model itself, resulting in discrepancies when compared to actual data.^[5,26]^ And the reliability of the epidemiological studies underpinning the GBD estimates for depression is poor.^[47]^ Our analysis relied on epidemiological data available from a subset of countries, which could introduce biases due to the methodological constraints inherent in the GBD 2021 framework. Such biases are likely to be more pronounced in low income nations, where challenges in data aggregation and potential under-reporting are more commonly encountered. These countries with limited primary data rely heavily on model-based estimates, which may compromise the accuracy and precision of the reported statistics. Additionally, cultural stigma and gendered under-reporting in regions such as Sub-Saharan Africa may further distort the data.^[48]^ Second, despite the employment of stringent statistical approaches within our study on WCBA suffering from DD, the variability in health data collection systems and reporting standards across different regions, particularly in low- and middle-income countries and those affected by conflict, and many countries with little or no access to treatment do not have proper data on DD,^[9]^ could contribute to gaps in the data and introduce biases. These factors might undermine the reliability of our results. Third, there is an acknowledged time lag associated with the disease burden figures utilized in this research. Fourth, due to space limitations, this paper does not provide a detailed comparison and analysis of the specific incidence and prevalence rates across various countries and provinces. Fifth, due to limitations inherent in the data, our study, while focused on WCBA, was unable to obtain data specific to pre- and postnatal periods and other fertility-related aspects. Consequently, an in-depth analysis of the relationship between childbirth and depression could not be conducted. Last, the estimates derived from our study are heavily dependent on the modeling techniques applied, indicating that the specific model selections and parameter configurations might significantly shape the study’s conclusions.

## 6. Conclusions

Between 1990 and 2021, there was a significant increase in the incidence, prevalence, and DALYs of depression among WCBA, particularly from 2020 to 2021. The predicted increase trends will continue globally at least until 2030. Persistent disparities existed between regions and nations. There was a significant correlation between the SDI and the AAPC in depression, with wealthier regions exhibiting greater increases. The age group spanning from 15 to 49 years also showed a slight increase, with minimal variation among different age groups within the same SDI regions. It is imperative for policymakers to assess their country-specific characteristics in relation to these disparities to make evidence-based decisions and understand their country’s standing in comparison with global benchmarks. In low-resource settings, scale up perinatal depression screening during pregnancy and the postpartum period. In high SDI countries, introduce workplace stress interventions such as flexible hours, confidential counseling, and digital cognitive behavioral therapy. Additionally, integrate brief mental health checks into reproductive health programs and train midwives and nurses in evidence-based psychological techniques, supported by tele-mentoring.

## Acknowledgments

We express our gratitude to the Institute for Health Metrics and Evaluation (IHME), the Global Burden of Disease (GBD) Collaborators, and the entire team of researchers and professionals who contributed the essential data utilized in the execution of this research endeavor.

## Author contributions

**Conceptualization:** Tiansheng Zhu, Xiayan Ye.

**Formal analysis:** Tiansheng Zhu, Ying Fan, Yunhan Shen.

**Investigation:** Tiansheng Zhu, Ying Fan, Ke Jin, Yunhan Shen, Xiayan Ye.

**Methodology:** Tiansheng Zhu, Xiayan Ye, Ying Fan, Yunhan Shen.

**Visualization:** Tiansheng Zhu, Ying Fan, Ke Jin, Yunhan Shen, Xiayan Ye.

**Writing – original draft:** Tiansheng Zhu.

**Writing – review & editing:** Tiansheng Zhu, Ying Fan, Ke Jin, Yunhan Shen, Xiayan Ye.

## Supplementary Material


