# Supplementary material for: Global, regional, and national burden and temporal trends of depressive disorders in women of child-bearing age, 1990 to 2021: A worldwide analysis
Source: Medicine (Baltimore). 2025 Oct 24;104(43):e45215. doi: 10.1097/MD.0000000000045215 (PMC12558287; doi:10.1097/MD.0000000000045215)
Supplement: Supplementary file 1 [file medi-104-e45215-s001.docx]

# Supplementary Table 1. The number, Age-standardized rate of prevalence for depressive disorders among WCBA in 1990 and 2021, and their temporal trends from 1990 to 2021.

| Variables | Prevalence in 1990  (95% UI) |  |  | Prevalence in 2021  (95% UI) | |  | AAPC  (95% CI) | | |
| --- | --- | --- | --- | --- | --- | --- | --- | --- | --- |
|  | Number(no.*10^3^)  (95% UI) | Age-standardized Rate per 100 000  (95% UI) |  | Number(no.* 10^3^)  (95% UI) | Age-standardized Rate per 100 000  (95% UI) | | |  | For rate  (95% CI) |
| **Global** | 72345.83  (57791.97 - 89932.62) | 5545.28  (4536.91 - 6714.57) |  | 121237.94  (96047.91 - 152522.49) | 6173.45  (5031.26 - 7547.03) | | |  | 0.38  (0.27 - 0.5) |
| **SDI** |  |  |  |  |  | | |  |  |
| High SDI | 13233.1  (10916.85 - 15950.1) | 5796.66  (4892.8 - 6834.36) |  | 18551.85  (14883.92 - 22977.92) | 7677.86  (6299.36 - 9314.57) | | |  | 1.02  (0.97 - 1.07) |
| High-middle SDI | 14308.99  (11526.67 - 17540.61) | 5218.03  (4363.64 - 6156.9) |  | 17375.91  (13800.96 - 21678.71) | 5475.98  (4481.01 - 6628.27) | | |  | 0.16  (0 - 0.33) |
| Middle SDI | 21577.11  (17180.99 - 26782.97) | 5051.06  (4186.15 - 6038.31) |  | 34248  (27294.08 - 42579.97) | 5421.62  (4478.14 - 6517.73) | | |  | 0.28  (0.17 - 0.39) |
| Low-middle SDI | 16217.07  (12627.53 - 20522.42) | 6257.67  (5079.06 - 7602.19) |  | 33062.3  (25812.93 - 42318.83) | 6628.44  (5472.53 - 8023.23) | | |  | 0.2  (0 - 0.39) |
| Low SDI | 6947.49  (5356.4 - 8940.84) | 6575.38  (5237.86 - 8167.81) |  | 17910.68  (13611.88 - 23067.29) | 6848.6  (5481.49 - 8369.16) | | |  | 0.15  (0.03 - 0.26) |
| **Subtypes** |  |  |  |  |  | | |  |  |
| MDD | 49642.77  (37047.74-65849.47) | 3773.27  (2886.32-4872.37) |  | 85643.35  (62470.48-114966.39) | 4373.94  (3281.17-5705.14) | | |  | 0.55  (0.41 - 0.69) |
| Dysthymia | 23630.14  (17155.83-31565.77) | 1845.05  (1370.46-2406.89) |  | 37264.83  (27290.5- 49665.11) | 1883.78  (1419.48-2438.06 | | |  | 0.05  (0.02- 0.09） |
| **Region** |  |  |  |  |  | | |  |  |
| Andean Latin America | 394.89  (295.65 - 523.28) | 4327.74  (3455.85 - 5415.94) |  | 939.18  (689.17 - 1261.82) | 5361.92  (4232.06 - 6775.74) | | |  | 0.8  (0.38 - 1.21) |
| Australasia | 407.83  (325.4 - 503.08) | 7599.59  (6459.88 - 8825.65) |  | 604.27  (449.02 - 803.27) | 8462.14  (6641.73 - 10682.7) | | |  | 0.4  (0.35 - 0.46) |
| Caribbean | 576.59  (435.4 - 752.33) | 6320.15  (5062.86 - 7788.16) |  | 786.81  (570.49 - 1071.01) | 6502.68  (5054.12 - 8247.38) | | |  | 0.15  (0.1 - 0.2) |
| Central Asia | 745.43  (571.38 - 964) | 4607.34  (3663.44 - 5752.58) |  | 1283.74  (965.34 - 1682.02) | 5231.16  (4386.85 - 6220.99) | | |  | 0.48  (0.41 - 0.54) |
| Central Europe | 1319.93  (1039.07 - 1659.22) | 4241.09  (3441.08 - 5177.13) |  | 1266.68  (987.76 - 1614.91) | 4677.25  (3782.32 - 5767.7) | | |  | 0.36  (0.29 - 0.42) |
| Central Latin America | 1775.28  (1366.31 - 2301.5) | 4465.04  (3585.56 - 5499.39) |  | 4314.39  (3286.06 - 5582.29) | 6293.78  (5006.97 - 7782.3) | | |  | 1.34  (1.07 - 1.6) |
| Central  Sub-Saharan Africa | 1062.1  (780.56 - 1423.34) | 8977.33  (7198.35 - 11052.85) |  | 2949.08  (2121.74 - 4037.27) | 9386.69  (8418.78 - 10578.52) | | |  | 0.19  (0.04 - 0.34) |
| East Asia | 15021.79  (12122.43 - 18400.59) | 4689.83  (4074 - 5337.04) |  | 13471.03  (10977.07 - 16341) | 3700  (3219.01 - 4211.74) | | |  | -0.77  (-0.9 - -0.65) |
| Eastern Europe | 2856.73  (2252.29 - 3606.39) | 5063.54  (4232.23 - 6071.25) |  | 3118.66  (2437.25 - 3945.27) | 6116.21  (5307.23 - 7102.82) | | |  | 0.61  (0.53 - 0.69) |
| Eastern Sub-Saharan Africa | 2861.72  (2214.63 - 3671.49) | 7087.75  (5689.94 - 8723.67) |  | 7582.73  (5752.88 - 9816.1) | 7444.18  (5971.96 - 9113.05) | | |  | 0.18  (0.13 - 0.23) |
| High-income Asia Pacific | 1597.49  (1307.98 - 1938.62) | 3508.02  (3021.85 - 4052.91) |  | 1605.27  (1294.43 - 1981.4) | 4293.05  (3620.71 - 5047.59) | | |  | 0.69  (0.61 - 0.76) |
| High-income North America | 5238.89  (4277.2 - 6352.28) | 7007.87  (6074.44 - 8034.14) |  | 8696.42  (7044.88 - 10654.97) | 10443.59  (9025.03 - 12060.59) | | |  | 1.4  (1.25 - 1.55) |
| North Africa and Middle East | 5563.73  (4231.17 - 7288.24) | 7378.92  (5810.69 - 9318.09) |  | 13296.22  (9812.76 - 17760.31) | 8318.57  (6377.64 - 10713.07) | | |  | 0.48  (0.39 - 0.58) |
| Oceania | 65.84  (49.26 - 87.08) | 4395.87  (3610.34 - 5349.94) |  | 154.06  (114.11 - 205.08) | 4495.59  (3715.66 - 5436.12) | | |  | 0.06  (0.01 - 0.11) |
| South Asia | 15167.95  (11954.26 - 18984.32) | 6252.32  (5270.65 - 7335.93) |  | 31198.61  (24618.88 - 39265.41) | 6390.39  (5591.94 - 7267.4) | | |  | 0.03  (-0.28 - 0.33) |
| Southeast Asia | 4437.17  (3476.63 - 5619.22) | 3875.31  (3153.81 - 4724.88) |  | 7959  (6238.35 - 10098.33) | 4294.28  (3500.38 - 5237.18) | | |  | 0.33  (0.32 - 0.35) |
| Southern Latin America | 711.12  (555.06 - 908.56) | 5733.18  (4720.02 - 6955.12) |  | 1128.26  (850.78 - 1473.21) | 6509.67  (5403.26 - 7759.19) | | |  | 0.56  (0.45 - 0.68) |
| Southern Sub-Saharan Africa | 797.95  (635.52 - 988.61) | 6388.08  (5547.59 - 7275.56) |  | 1662.48  (1314.16 - 2086.08) | 7672.13  (6691.63 - 8756.91) | | |  | 0.71  (0.53 - 0.9) |
| Tropical Latin America | 2589.37  (2039.98 - 3260.98) | 6672.9  (5674.48 - 7812.79) |  | 4726.76  (3693.42 - 5948.77) | 7633.97  (6902.86 - 8500.56) | | |  | 0.63  (0.35 - 0.91) |
| Western Europe | 6682.64  (5519 - 8062.33) | 6951.88  (5848.28 - 8250.02) |  | 7757.61  (6002.39 - 9979.26) | 8254.89  (6545.12 - 10412.88) | | |  | 0.72  (0.56 - 0.88) |
| Western Sub-Saharan Africa | 2471.39  (1907.76 - 3177.81) | 6101.55  (4935.1 - 7489.96) |  | 6736.69  (5165.59 - 8681.68) | 5969.48  (4896.07 - 7200.13) | | |  | -0.04  (-0.19 - 0.1) |

WCBA, women of childbearing age

# Supplementary Table 2. The number, Age-standardized rate of Incidence for depressive disorders among WCBA in 1990 and 2021, and their temporal trends from 1990 to 2021.

| Variables | Incidence in 1990  (95% UI) |  |  | Incidence in 2021  (95% UI) | |  | AAPC  (95% CI) | | |
| --- | --- | --- | --- | --- | --- | --- | --- | --- | --- |
|  | Number(no.*10^3^)  (95% UI) | Age-standardized Rate per 100 000  (95% UI) |  | Number(no.* 10^3^)  (95% UI) | Age-standardized Rate per 100 000  (95% UI) | | |  | For rate  (95% CI) |
| **Global** | 77722.84  (58860.49 - 102539.34) | 5898.5  (4576.93 - 7577.55) |  | 133248.59  (99032.45 - 177876.46) | 6808.01  (5203.36 - 8831.4) | | |  | 0.53  (0.38 - 0.67) |
| **SDI** |  |  |  |  |  | | |  |  |
| High SDI | 14095.7  (11172.41 - 17813.54) | 6219.44  (5041.64 - 7678.41) |  | 21662.49  (16621.41 - 28113.42) | 9099.46  (7133.58 - 11531.35) | | |  | 1.4  (1.25 - 1.55) |
| High-middle SDI | 14966.53  (11469.34 - 19395.26) | 5424.7  (4317.33 - 6760.12) |  | 17857.23  (13115.94 - 23816.97) | 5778.86  (4373.43 - 7473.41) | | |  | 0.23  (0.01 - 0.45) |
| Middle SDI | 22649.07  (16997.95 - 29922.16) | 5183.69  (4048.51 - 6596.13) |  | 36081.48  (26891.51 - 47657.46) | 5767.56  (4453.41 - 7365.35) | | |  | 0.43  (0.29 - 0.57) |
| Low-middle SDI | 18391.51  (13457.05 - 25013.2) | 7035.36  (5371.49 - 9174.4) |  | 37747.94  (27469.87 - 51290.42) | 7551.4  (5816.46 - 9697) | | |  | 0.24  (-0.03 -0.52) |
| Low SDI | 7553.85  (5354.32 - 10472.43) | 7097.66  (5220.18 - 9487.32) |  | 19801.19  (13890.12 - 27443.21) | 7509.35  (5570.07 - 9860.99) | | |  | 0.16  (-0.14 0.45) |
| **Subtypes** |  |  |  |  |  | | |  |  |
| MDD | 73623.31  (54839.09 - 98562.34) | 5587.09  (4267.75 - 7279.58) |  | 127019.29  (93094.22 - 171543.48) | 6490.04  (4890.88 - 8518.22) | | |  | 0.55  (0.40 - 0.7) |
| Dysthymia | 4099.53  (2677.26 - 5792.35) | 311.41  (207.6 - 430.16) |  | 6229.31  (4023.8 - 8756.31) | 317.97  (211.57 - 433.79) | | |  | 0.06  (0.03 - 0.09) |
| **Region** |  |  |  |  |  | | |  |  |
| Andean Latin America | 438.67  (303.75 - 617.43) | 4737  (3499.51 - 6295.29) |  | 1105.34  (751.1 - 1589.97) | 6320.57  (4617.53 - 8553.11) | | |  | 1.09  (0.52 - 1.67) |
| Australasia | 480  (369.33 - 616.43) | 8977.64  (7359.06 - 10846.97) |  | 723.16  (498.99 - 1010.53) | 10269.64  (7490.52 - 13583.46) | | |  | 0.5  (0.43 - 0.56) |
| Caribbean | 708.02  (507.84 - 969.85) | 7697.77  (5857.85 - 9967.13) |  | 969.57  (658.74 - 1384.73) | 8029.7  (5846.73 - 10683.77) | | |  | 0.21  (0.14 - 0.28) |
| Central Asia | 762.53  (544.49 - 1049.48) | 4676.28  (3478.14 - 6190.8) |  | 1367.57  (948.06 - 1922.3) | 5623.36  (4354 - 7164.73) | | |  | 0.7  (0.6 - 0.8) |
| Central Europe | 1272.97  (942.66 - 1698.35) | 4115.36  (3142.57 - 5334.04) |  | 1273.33  (914.04 - 1725.19) | 4795.85  (3556.81 - 6329.03) | | |  | 0.56  (0.45 - 0.66) |
| Central Latin America | 2056.46  (1474.4 - 2831.11) | 5119.69  (3836.95 - 6706.71) |  | 5395.96  (3919.15 - 7296.87) | 7882.55  (5977.91 - 10188.15) | | |  | 1.69  (1.36 - 2.01) |
| Central Sub-Saharan Africa | 1276.62  (879.91 - 1821.33) | 10640.71  (8018.57 - 13968.32) |  | 3584.29  (2373.08 - 5212.81) | 11270.02  (9313.86 - 13481.74) | | |  | 0.26  (0.05 - 0.47) |
| East Asia | 14573.7  (11063.94 - 19051.3) | 4412.52  (3605.93 - 5350.42) |  | 10395.45  (7909.21 - 13367.18) | 2956.12  (2394.16 - 3581.9) | | |  | -1.16  (-1.54 -0.77) |
| Eastern Europe | 3022.73  (2188.45 - 4091.17) | 5392.25  (4144.45 - 6914.9) |  | 3496.27  (2499.38 - 4757.67) | 6990.81  (5546.81 - 8695.87) | | |  | 0.84  (0.72 - 0.97) |
| Eastern Sub-Saharan Africa | 2992.87  (2132.52 - 4133.29) | 7375.7  (5483.53 - 9746.21) |  | 8187.85  (5707.05 - 11439.4) | 7985.77  (5902.77 - 10515.46) | | |  | 0.3  (0.23 - 0.37) |
| High-income Asia Pacific | 1741.96  (1355 - 2240.8) | 3838.31  (3138.06 - 4702.12) |  | 1832.83  (1393.44 - 2364.64) | 5010.7  (3974.29 - 6163.55) | | |  | 0.92  (0.78 - 1.06) |
| High-income North America | 5275.31  (4101.57 - 6757.93) | 7145.82  (5901.07 - 8643.76) |  | 10479.47  (8142.16 - 13299.66) | 12689.95  (10517.4 - 15164.01) | | |  | 1.99  (1.53 - 2.44) |
| North Africa and Middle East | 6585.65  (4704.06 - 9116.66) | 8629.46  (6390.3 - 11534.04) |  | 16021.22  (10983.02 - 22831.95) | 10032.79  (7148.67 - 13778.78) | | |  | 0.63  (0.33 - 0.93) |
| Oceania | 62.23  (43.09 - 88.31) | 3968.59  (3024.6 - 5155.86) |  | 143.68  (95.17 - 209.65) | 4125.32  (3050.51 - 5468.24) | | |  | 0.1  (0.01 - 0.19) |
| South Asia | 17346.56  (12898.19 - 23184.71) | 7116.76  (5668.13 - 8907.15) |  | 35575.99  (26421.85 - 47285.18) | 7283.76  (6003.1 - 8742.38) | | |  | 0.02  (-0.41 -0.45) |
| Southeast Asia | 3802.11  (2781.51 - 5120.36) | 3196.08  (2431.96 - 4130.24) |  | 7017.74  (5014.07 - 9561.43) | 3829.96  (2844.28 - 5016.8) | | |  | 0.59  (0.56 - 0.62) |
| Southern Latin America | 868.79  (655.86 - 1148.7) | 6983.96  (5560.06 - 8773.67) |  | 1401.35  (1012.96 - 1899.05) | 8133.97  (6475.99 - 10050.7) | | |  | 0.62  (0.25 - 1) |
| Southern Sub-Saharan Africa | 851.74  (638.67 - 1125.69) | 6761.59  (5540.36 - 8208.39) |  | 1889.7  (1403.73 - 2506.39) | 8722.61  (7152.71 - 10514.12) | | |  | 0.99  (0.72 - 1.27) |
| Tropical Latin America | 3243.57  (2443.73 - 4270.77) | 8304.3  (6759.81 - 10166.14) |  | 6004.24  (4494.35 - 7859.87) | 9756.89  (8444.5 - 11297) | | |  | 0.75  (0.39 - 1.12) |
| Western Europe | 7776.73  (6187.7 - 9786.38) | 8121.24  (6576.4 - 10044.53) |  | 9415.89  (6929.83 - 12706.63) | 10094.71  (7592.21 - 13348.43) | | |  | 0.9  (0.71 - 1.08) |
| Western Sub-Saharan Africa | 2583.6  (1839.83 - 3578.08) | 6321.17  (4728.67 - 8339.34) |  | 6967.69  (4926.01 - 9745.6) | 6114.54  (4636.18 - 7982.09) | | |  | -0.01  (-0.31 -0.29) |

# Supplementary table 3. ASIR (per100,000) of WCBA due to depressive disorders in 204 countries and territories in 2021 (ordered by value).

| **Location** | **Value** | **Upper** | **Lower** |
| --- | --- | --- | --- |
| Myanmar | 2717.484 | 3758.258 | 1921.733 |
| China | 2947.483 | 3556.74 | 2396.504 |
| Taiwan (Province of China) | 3058.112 | 4038.753 | 2266.531 |
| Democratic People's Republic of Korea | 3255.018 | 4130.849 | 2547.733 |
| Lao People's Democratic Republic | 3293.296 | 4477.086 | 2409.175 |
| Brunei Darussalam | 3383.89 | 4652.219 | 2445.855 |
| American Samoa | 3386.912 | 4728.61 | 2380.101 |
| Colombia | 3409.81 | 4651.029 | 2431.368 |
| Poland | 3416.861 | 4251.382 | 2677.78 |
| Tonga | 3547.693 | 4842.391 | 2585.599 |
| Viet Nam | 3611.829 | 4972.718 | 2572.096 |
| Samoa | 3630.01 | 4905.857 | 2631.839 |
| Indonesia | 3651.765 | 4658.078 | 2828.12 |
| Kiribati | 3727.57 | 4993.396 | 2752.524 |
| Micronesia (Federated States of) | 3756.488 | 4873.102 | 2815.066 |
| Northern Mariana Islands | 3773.038 | 5283.668 | 2627.034 |
| Seychelles | 3800.174 | 5080.605 | 2724.065 |
| Marshall Islands | 3802.648 | 4977.681 | 2829.777 |
| Thailand | 3877.74 | 5060.413 | 2968.659 |
| Timor-Leste | 3924.637 | 5287.702 | 2852.924 |
| Vanuatu | 4111.517 | 5746.297 | 2875.618 |
| Papua New Guinea | 4124.729 | 5492.815 | 3067.239 |
| Solomon Islands | 4193.918 | 5506.525 | 3227.967 |
| Singapore | 4201.222 | 5596.515 | 3100.776 |
| Fiji | 4334.151 | 5816.397 | 3161.728 |
| Philippines | 4480.85 | 5528.257 | 3579.85 |
| Cambodia | 4485.278 | 5739.321 | 3448.675 |
| Republic of Korea | 4521.323 | 5950.101 | 3311.304 |
| Guam | 4543.96 | 6244.999 | 3277.911 |
| Tuvalu | 4556.943 | 6273.332 | 3175.784 |
| Tokelau | 4603.126 | 6794.521 | 3105.788 |
| Niue | 4604.143 | 6405.246 | 3247.155 |
| Nauru | 4611.018 | 6517.106 | 3223.512 |
| Palau | 4626.496 | 6631.051 | 3155.331 |
| Cook Islands | 4628.322 | 6731.292 | 3098.12 |
| Sri Lanka | 4659.68 | 5981.29 | 3563.477 |
| Malaysia | 4749.722 | 6343.377 | 3519.765 |
| Peru | 4791.786 | 6598.999 | 3357.797 |
| Maldives | 4893.219 | 6498.747 | 3632.866 |
| Hungary | 5083.24 | 6531.439 | 3766.423 |
| Zimbabwe | 5148.416 | 6957.269 | 3741.697 |
| Puerto Rico | 5188.719 | 7361.88 | 3536.286 |
| Azerbaijan | 5198.018 | 6858.472 | 3762.699 |
| Nigeria | 5211.873 | 6341.551 | 4222.381 |
| Mauritania | 5227.112 | 7070.229 | 3795.399 |
| Tajikistan | 5232.754 | 6887.246 | 3840.908 |
| Albania | 5277.308 | 7086.527 | 3914.177 |
| Romania | 5298.938 | 6975.842 | 3928.342 |
| Japan | 5301.71 | 6435.383 | 4372.699 |
| Serbia | 5346.198 | 7190.782 | 3967.033 |
| Uzbekistan | 5359.027 | 6284.99 | 4859.123 |
| Turkmenistan | 5372.263 | 7390.364 | 3810.573 |
| Croatia | 5421.745 | 7147.089 | 4086.261 |
| Armenia | 5478.106 | 7416.927 | 3954.161 |
| Mali | 5498.159 | 7365.114 | 4194.284 |
| North Macedonia | 5686.533 | 7145.444 | 4420.31 |
| Czechia | 5708.579 | 7782.155 | 4006.525 |
| Slovakia | 5749.758 | 8035.301 | 4053.669 |
| Georgia | 5874.035 | 8025.578 | 4112.032 |
| Bosnia and Herzegovina | 5889.633 | 7948.588 | 4133.699 |
| Côte d'Ivoire | 5891.693 | 7865.852 | 4281.889 |
| Venezuela (Bolivarian Republic of) | 6080.453 | 8216.711 | 4488.01 |
| Bulgaria | 6120.751 | 8380.881 | 4405.05 |
| Montenegro | 6178.858 | 8091.948 | 4613.22 |
| Kazakhstan | 6190.953 | 8205.861 | 4548.937 |
| Bhutan | 6236.297 | 8668.625 | 4359.206 |
| Sao Tome and Principe | 6264.098 | 8472.831 | 4495.394 |
| Slovenia | 6308.256 | 8603.581 | 4561.848 |
| Republic of Moldova | 6324.898 | 8196.318 | 4928.644 |
| Kyrgyzstan | 6360.503 | 8121.536 | 4898.679 |
| Russian Federation | 6361.073 | 7710.55 | 5207.099 |
| Iceland | 6380.195 | 8760.419 | 4479.528 |
| Burkina Faso | 6407.146 | 8924.291 | 4507.569 |
| Niger | 6471.061 | 8596.618 | 4777.121 |
| Senegal | 6525.305 | 8874.309 | 4788.776 |
| Panama | 6560.248 | 8963.67 | 4757.45 |
| Mongolia | 6569.037 | 9009.947 | 4738.431 |
| Zambia | 6663.726 | 8722.513 | 5019.613 |
| Antigua and Barbuda | 6740.216 | 9212.484 | 4759.888 |
| Argentina | 6744.086 | 8169.828 | 5541.481 |
| Malawi | 6750.936 | 9496.225 | 4752.968 |
| India | 6820.945 | 7950.894 | 5823.065 |
| Grenada | 6865.953 | 9133.196 | 4939.691 |
| Comoros | 6884.569 | 8806.128 | 5296.899 |
| Dominica | 6889.168 | 9341.708 | 4901.464 |
| Belize | 6906.328 | 9237.563 | 5115.871 |
| United States Virgin Islands | 6931.319 | 9702.006 | 4736.048 |
| Bahamas | 7015.002 | 9566.32 | 5078.254 |
| Togo | 7031.187 | 9185.637 | 5242.463 |
| Jamaica | 7060.545 | 9782.057 | 4925.482 |
| Ethiopia | 7061.363 | 8521.336 | 5929.81 |
| Bermuda | 7062.484 | 9817.616 | 4775.038 |
| Saint Vincent and the Grenadines | 7082.983 | 9628.331 | 5072.283 |
| Sierra Leone | 7101.627 | 9766.211 | 5118.649 |
| Ghana | 7137.407 | 9583.076 | 5126.205 |
| Barbados | 7171.97 | 9742.167 | 5144.668 |
| Benin | 7187.461 | 9720.696 | 5271.463 |
| Costa Rica | 7234.435 | 9757.325 | 5208.905 |
| Honduras | 7256.814 | 9892.567 | 5155.709 |
| Austria | 7277.523 | 9818.251 | 5288.705 |
| Namibia | 7330.273 | 9953.22 | 5446.549 |
| Djibouti | 7351.609 | 9576.347 | 5476.747 |
| Malta | 7392.996 | 10042.41 | 5243.112 |
| Nicaragua | 7424.668 | 9523.812 | 5705.55 |
| Kenya | 7469.656 | 9219.766 | 5981.269 |
| Pakistan | 7492.716 | 9576.491 | 5663.231 |
| Luxembourg | 7499.555 | 9796.252 | 5566.376 |
| Saint Lucia | 7524.262 | 10121.18 | 5481.855 |
| Cameroon | 7532.162 | 9712.634 | 5727.315 |
| Cyprus | 7549.474 | 10306.14 | 5377.826 |
| Guinea | 7618.437 | 10376.32 | 5515.577 |
| United Republic of Tanzania | 7641.619 | 10228.29 | 5522.729 |
| South Sudan | 7674.606 | 10288.84 | 5846.163 |
| Eritrea | 7719.672 | 8829.03 | 7154.338 |
| Ecuador | 7747.17 | 10196.33 | 5823.894 |
| Estonia | 7753.191 | 10636.26 | 5497.987 |
| Madagascar | 7814.609 | 10835.95 | 5404.454 |
| Guinea-Bissau | 7824.749 | 10400.92 | 5829.134 |
| Kuwait | 7980.211 | 10839.98 | 5696.137 |
| Cuba | 8038.921 | 10725.7 | 5914.216 |
| Latvia | 8041.18 | 11069.62 | 5609.323 |
| Mauritius | 8044.694 | 10549.26 | 6003.175 |
| Burundi | 8064.428 | 10821.82 | 5962.862 |
| El Salvador | 8065.428 | 10462.38 | 6215.398 |
| Haiti | 8115.463 | 10284.41 | 6201.5 |
| Dominican Republic | 8246.797 | 11151.22 | 5986.121 |
| New Zealand | 8307.462 | 10798.97 | 6414.488 |
| Mozambique | 8365.67 | 11712.75 | 5865.249 |
| Germany | 8403.431 | 11339.23 | 6079.151 |
| Ukraine | 8414.91 | 10152.45 | 6973.041 |
| United Arab Emirates | 8526.539 | 11201.68 | 6557.809 |
| Cabo Verde | 8533.651 | 11499.05 | 6205.868 |
| Iraq | 8548.054 | 9969.317 | 7764.261 |
| Denmark | 8551.739 | 11566.39 | 6082.47 |
| Guatemala | 8574.383 | 12074.8 | 5985.16 |
| Chad | 8575.242 | 11156.76 | 6382.403 |
| Saint Kitts and Nevis | 8676.35 | 11711.52 | 6238.855 |
| Botswana | 8683.773 | 11528.57 | 6468.122 |
| Algeria | 8896.837 | 12015.13 | 6547.891 |
| Bolivia (Plurinational State of) | 8903.551 | 11805.41 | 6574.672 |
| Belgium | 8949.293 | 12014.91 | 6565.779 |
| Egypt | 8949.981 | 12444.56 | 6158.061 |
| Uruguay | 8980.007 | 11644.83 | 6728.604 |
| Norway | 9022.033 | 11282.98 | 6951.366 |
| Liberia | 9029.017 | 11772.4 | 6862.201 |
| Saudi Arabia | 9053.126 | 12104.94 | 6571.654 |
| Paraguay | 9064.104 | 11452.17 | 7078.359 |
| Sudan | 9078.981 | 12065.77 | 6785.754 |
| Canada | 9096.416 | 12370.44 | 6485.362 |
| Syrian Arab Republic | 9123.933 | 12149.43 | 6938.55 |
| Qatar | 9152.24 | 13071.39 | 6123.422 |
| Netherlands | 9183.953 | 12322.17 | 6532.271 |
| Switzerland | 9228.465 | 12361.42 | 6664.961 |
| Somalia | 9310.926 | 11192.78 | 8188.923 |
| Oman | 9332.76 | 13424.73 | 6195.804 |
| Rwanda | 9386.638 | 12482.21 | 6789.481 |
| Andorra | 9421.871 | 13076.91 | 6508.15 |
| South Africa | 9495.575 | 11068 | 8148.865 |
| Belarus | 9514.304 | 12216.85 | 7218.595 |
| Lithuania | 9518.643 | 12643.93 | 6925.304 |
| Libya | 9530.675 | 12312.95 | 7134.036 |
| Jordan | 9579.584 | 13132.04 | 6734.538 |
| Trinidad and Tobago | 9624.698 | 12951.27 | 7147.836 |
| Brazil | 9772.366 | 11264.5 | 8533.527 |
| Turkey | 9883.899 | 14194.98 | 6684.58 |
| Italy | 9911.364 | 12517.33 | 7687.995 |
| Israel | 9945.828 | 13063.44 | 7285.854 |
| Mexico | 9961.295 | 12328.41 | 7972.676 |
| France | 10135.1 | 13389.24 | 7474.78 |
| Yemen | 10178.37 | 12998.21 | 7625.389 |
| Bangladesh | 10285.19 | 13380.68 | 7771.168 |
| United Kingdom | 10342.25 | 13311.45 | 7907.782 |
| Nepal | 10351.69 | 13621.22 | 7594.273 |
| Eswatini | 10352.94 | 14079.8 | 7468.664 |
| Australia | 10655.35 | 14237.38 | 7590.959 |
| Democratic Republic of the Congo | 10731.63 | 12061.78 | 9879.9 |
| Monaco | 10882.45 | 15683.64 | 7279.396 |
| Bahrain | 10918.27 | 15224.06 | 7527.825 |
| San Marino | 11099.34 | 15562.23 | 7658.178 |
| Finland | 11190.88 | 14724.89 | 8312.305 |
| Gabon | 11365.03 | 15090.08 | 8274.853 |
| Chile | 11524.79 | 15557.04 | 8207.012 |
| Ireland | 11604.98 | 15332.8 | 8480.275 |
| Morocco | 11687.82 | 15646.15 | 8343.331 |
| Uganda | 11746.52 | 15746.95 | 8430.343 |
| Afghanistan | 11768.52 | 13226.04 | 11018.82 |
| Central African Republic | 11824.85 | 15331.35 | 9280.517 |
| Equatorial Guinea | 12062.3 | 16480.41 | 8594.696 |
| Congo | 12064.56 | 15893.96 | 9076.425 |
| Sweden | 12110.76 | 15101.18 | 9460.214 |
| Gambia | 12222.79 | 16352.01 | 8834.383 |
| Iran (Islamic Republic of) | 12267.94 | 15439.98 | 9640.113 |
| Angola | 12452.72 | 16729.87 | 9000.175 |
| Spain | 12605.74 | 16651.14 | 9240.605 |
| Suriname | 12614.94 | 16446.59 | 9447.044 |
| Tunisia | 12741.92 | 17543.76 | 8901.247 |
| Palestine | 12928.12 | 18080.74 | 8935.622 |
| Lebanon | 12984.07 | 17165.16 | 9614.212 |
| Portugal | 13053.77 | 17399.54 | 9620.935 |
| United States of America | 13072.89 | 15446.03 | 10947.31 |
| Guyana | 13100.09 | 17125.8 | 9677.525 |
| Greece | 13841.31 | 18737.16 | 9964.977 |
| Lesotho | 13845.53 | 18398.96 | 10057.92 |
| Greenland | 20221.09 | 26364.71 | 15099.65 |

ASIR, Age-standardized incidence rate; WCBA, women of childbearing age;

# Supplementary table 4. ASPR (per100,000) of WCBA due to depressive disorders in 204 countries and territories in 2021 (ordered by value).

| **Location** | **Val** | **Upper** | **Lower** |
| --- | --- | --- | --- |
| Brunei Darussalam | 3155.589912 | 3934.251959 | 2549.677733 |
| Colombia | 3221.496575 | 3990.409975 | 2552.045653 |
| Myanmar | 3547.395768 | 4372.776617 | 2888.32678 |
| Singapore | 3606.556469 | 4553.900966 | 2848.080453 |
| China | 3695.549772 | 4185.971949 | 3234.051934 |
| Taiwan (Province of China) | 3733.868227 | 4535.016025 | 3027.051454 |
| Poland | 3768.224456 | 4330.079947 | 3297.061003 |
| Democratic People's Republic of Korea | 3860.682462 | 4498.318176 | 3356.916351 |
| Lao People's Democratic Republic | 3949.063895 | 4837.3172 | 3162.320526 |
| American Samoa | 3996.431413 | 5015.282327 | 3203.555437 |
| Republic of Korea | 4026.538426 | 5002.640659 | 3199.419599 |
| Tonga | 4107.897891 | 5053.715605 | 3340.594755 |
| Viet Nam | 4154.226978 | 5174.853008 | 3294.18803 |
| Samoa | 4162.412119 | 5108.767749 | 3356.892923 |
| Indonesia | 4171.846144 | 4843.732354 | 3588.895455 |
| Kiribati | 4227.309107 | 5103.155535 | 3475.892791 |
| Micronesia (Federated States of) | 4247.191831 | 5009.814153 | 3527.75794 |
| Northern Mariana Islands | 4251.56278 | 5389.340063 | 3312.843979 |
| Marshall Islands | 4276.878244 | 5119.109496 | 3607.65311 |
| Seychelles | 4287.925859 | 5178.667935 | 3540.146922 |
| Peru | 4326.452181 | 5456.626947 | 3403.798199 |
| Thailand | 4331.09361 | 5106.915132 | 3634.815756 |
| Timor-Leste | 4372.588381 | 5372.489349 | 3529.677989 |
| Japan | 4470.528212 | 5149.94637 | 3893.35451 |
| Vanuatu | 4485.316966 | 5681.311366 | 3504.599023 |
| Papua New Guinea | 4495.90799 | 5363.672005 | 3784.435045 |
| Solomon Islands | 4537.544233 | 5403.49972 | 3885.114667 |
| Puerto Rico | 4597.243126 | 6039.022759 | 3499.121836 |
| Fiji | 4632.309989 | 5692.598809 | 3727.610282 |
| Philippines | 4717.284487 | 5354.836335 | 4200.066075 |
| Cambodia | 4749.019258 | 5509.570094 | 4038.44195 |
| Guam | 4779.60911 | 6020.9268 | 3845.292477 |
| Tuvalu | 4779.751658 | 5890.505767 | 3837.342294 |
| Tokelau | 4810.254742 | 6180.361676 | 3737.257904 |
| Niue | 4811.171848 | 5992.623369 | 3908.059391 |
| Nauru | 4816.41218 | 6002.580058 | 3883.208657 |
| Palau | 4826.810112 | 6215.813098 | 3809.493926 |
| Cook Islands | 4829.227916 | 6260.742058 | 3742.771763 |
| Hungary | 4858.83644 | 5828.672006 | 4022.760747 |
| Sri Lanka | 4880.87333 | 5791.960883 | 4147.234846 |
| Malaysia | 4926.062765 | 6012.610289 | 4118.976809 |
| Azerbaijan | 4936.885798 | 5868.580235 | 4056.808241 |
| Tajikistan | 4963.109383 | 5968.55528 | 4102.907553 |
| Romania | 5007.588747 | 6068.415484 | 4099.722733 |
| Albania | 5007.924884 | 6095.448401 | 4167.414536 |
| Maldives | 5022.423855 | 6150.236035 | 4174.118202 |
| Serbia | 5038.120469 | 6085.814284 | 4224.250828 |
| Uzbekistan | 5055.670734 | 5420.510178 | 5051.014461 |
| Turkmenistan | 5060.378701 | 6311.452022 | 4075.134977 |
| Venezuela (Bolivarian Republic of) | 5081.864467 | 6407.953562 | 4063.054409 |
| Croatia | 5091.082104 | 6134.741081 | 4194.425009 |
| Armenia | 5121.938668 | 6232.899959 | 4185.492352 |
| North Macedonia | 5268.121846 | 6137.613873 | 4587.791469 |
| Czechia | 5283.082184 | 6613.026126 | 4120.570425 |
| Slovakia | 5302.248395 | 6728.884247 | 4165.02461 |
| Zimbabwe | 5310.395328 | 6432.473965 | 4340.434761 |
| Mauritania | 5369.968751 | 6487.383815 | 4358.258192 |
| Nigeria | 5370.107315 | 6034.674516 | 4768.224097 |
| Georgia | 5382.958733 | 6732.291918 | 4262.326673 |
| Panama | 5395.965495 | 6898.622112 | 4275.508361 |
| Bosnia and Herzegovina | 5407.2106 | 6747.667803 | 4331.895761 |
| Bulgaria | 5548.216578 | 6971.263103 | 4438.189203 |
| Argentina | 5550.912268 | 6421.605936 | 4763.9447 |
| Mali | 5556.050749 | 6623.683387 | 4710.793034 |
| Montenegro | 5594.835425 | 6769.452489 | 4586.24607 |
| Kazakhstan | 5622.326368 | 6932.354089 | 4474.540801 |
| Antigua and Barbuda | 5631.914196 | 7170.819034 | 4446.747032 |
| Republic of Moldova | 5658.668027 | 6788.549201 | 4869.714959 |
| Bhutan | 5671.3284 | 7116.271751 | 4459.904939 |
| Slovenia | 5689.236274 | 7230.967527 | 4503.081959 |
| Russian Federation | 5707.722019 | 6463.41679 | 5134.721656 |
| Grenada | 5719.567416 | 7137.673737 | 4554.16385 |
| Kyrgyzstan | 5720.34754 | 6730.708569 | 4882.715737 |
| Dominica | 5728.755247 | 7232.347891 | 4513.432997 |
| Belize | 5750.784378 | 7120.157063 | 4654.394981 |
| United States Virgin Islands | 5768.451972 | 7608.66068 | 4362.553008 |
| Iceland | 5775.335185 | 7228.331921 | 4529.273277 |
| Bahamas | 5811.593453 | 7336.958783 | 4623.165328 |
| C么te d'Ivoire | 5818.121812 | 7040.565264 | 4716.577403 |
| Jamaica | 5842.129103 | 7500.936929 | 4520.3583 |
| Honduras | 5847.546597 | 7548.061164 | 4475.412941 |
| Costa Rica | 5853.768322 | 7492.691175 | 4577.071354 |
| Bermuda | 5853.935123 | 7547.110654 | 4405.813518 |
| Saint Vincent and the Grenadines | 5864.128579 | 7393.882815 | 4619.694583 |
| Mongolia | 5876.911613 | 7440.330179 | 4643.318811 |
| Barbados | 5921.619546 | 7422.012195 | 4712.316984 |
| Nicaragua | 6002.922754 | 7236.466958 | 4997.500576 |
| Sao Tome and Principe | 6075.639687 | 7470.022842 | 4927.997203 |
| India | 6089.458551 | 6697.752812 | 5514.235599 |
| Saint Lucia | 6155.705354 | 7738.991988 | 4907.286426 |
| Burkina Faso | 6163.960446 | 7848.44026 | 4848.062357 |
| Niger | 6210.133428 | 7479.37739 | 5096.709165 |
| Senegal | 6228.018124 | 7686.83696 | 5121.097239 |
| Ecuador | 6352.780295 | 7767.3421 | 5147.113437 |
| Austria | 6358.809116 | 8092.116747 | 5026.739816 |
| El Salvador | 6439.689066 | 7894.464444 | 5367.015502 |
| Malta | 6460.130079 | 8040.521494 | 5081.571955 |
| Cuba | 6489.067262 | 8166.32601 | 5177.937998 |
| Pakistan | 6517.313268 | 7769.429815 | 5396.708134 |
| Luxembourg | 6519.65463 | 8008.616586 | 5147.465631 |
| Zambia | 6563.265676 | 7781.737611 | 5520.581409 |
| Cyprus | 6564.334871 | 8149.768171 | 5274.4267 |
| Haiti | 6571.488048 | 7850.650865 | 5454.175707 |
| Togo | 6578.963871 | 7852.18154 | 5494.738309 |
| Estonia | 6615.418341 | 8488.157948 | 5181.190461 |
| Sierra Leone | 6629.055514 | 8225.493714 | 5318.380933 |
| Malawi | 6629.249932 | 8407.542464 | 5224.897798 |
| Ghana | 6650.315217 | 8160.003005 | 5419.690562 |
| Dominican Republic | 6654.057857 | 8417.7842 | 5254.668174 |
| Benin | 6679.723317 | 8189.184209 | 5383.894884 |
| Comoros | 6712.160216 | 7781.114345 | 5867.441808 |
| Namibia | 6751.536775 | 8278.938298 | 5634.744241 |
| Guatemala | 6760.511332 | 9072.882877 | 5037.253283 |
| Latvia | 6786.376031 | 8678.21338 | 5172.02893 |
| Ethiopia | 6820.358456 | 7577.980856 | 6341.81185 |
| Cameroon | 6911.447151 | 8157.177325 | 5903.873367 |
| Saint Kitts and Nevis | 6928.159605 | 8833.714871 | 5454.919057 |
| Kuwait | 6941.62997 | 8744.90437 | 5536.529023 |
| Guinea | 6965.237864 | 8599.158329 | 5604.50132 |
| Germany | 6976.314347 | 8874.88803 | 5470.919223 |
| Ukraine | 7034.410471 | 7979.76546 | 6363.635585 |
| Djibouti | 7035.718858 | 8332.709866 | 5908.836607 |
| Uruguay | 7051.843971 | 8728.808363 | 5579.206251 |
| Kenya | 7078.978393 | 8145.639579 | 6081.988863 |
| Bolivia (Plurinational State of) | 7081.786464 | 8882.644645 | 5605.7908 |
| Guinea-Bissau | 7102.198762 | 8619.850497 | 5885.385083 |
| New Zealand | 7170.461969 | 8707.808109 | 5977.365851 |
| Denmark | 7208.714499 | 9143.447246 | 5603.237307 |
| Mauritius | 7219.253035 | 8854.402413 | 5832.535993 |
| United Republic of Tanzania | 7220.834667 | 8755.797734 | 5905.744362 |
| Paraguay | 7246.511895 | 8698.362358 | 6111.239965 |
| South Sudan | 7251.932192 | 8728.808569 | 6197.803991 |
| Eritrea | 7272.311524 | 7494.395625 | 7553.218036 |
| United Arab Emirates | 7275.734444 | 8853.745245 | 6152.146935 |
| Iraq | 7296.480173 | 7943.197535 | 7277.675404 |
| Belgium | 7323.574282 | 9239.76576 | 5793.319069 |
| Madagascar | 7337.402845 | 9243.491644 | 5706.647946 |
| Burundi | 7498.070127 | 9106.582636 | 6258.527415 |
| Algeria | 7546.093244 | 9393.965368 | 6207.688067 |
| Cabo Verde | 7559.99132 | 9325.848919 | 6033.209902 |
| Netherlands | 7570.255427 | 9604.915144 | 5858.991574 |
| Egypt | 7572.915551 | 9832.868775 | 5753.744583 |
| Trinidad and Tobago | 7588.871693 | 9710.447799 | 5995.919774 |
| Norway | 7608.042446 | 9015.086296 | 6320.720063 |
| Chad | 7618.460138 | 9269.749511 | 6262.700806 |
| Brazil | 7641.526019 | 8465.14591 | 6975.469502 |
| Saudi Arabia | 7648.158407 | 9463.212629 | 6180.028672 |
| Botswana | 7665.489165 | 9372.793119 | 6240.126823 |
| Sudan | 7666.661244 | 9540.107677 | 6286.463977 |
| Mozambique | 7694.957463 | 9833.953831 | 6004.627925 |
| Syrian Arab Republic | 7698.291366 | 9527.210391 | 6434.794226 |
| Mexico | 7715.06822 | 9155.487621 | 6419.970205 |
| Qatar | 7725.643214 | 10279.31824 | 5749.48519 |
| Belarus | 7778.957852 | 9435.788247 | 6388.144561 |
| Lithuania | 7787.358062 | 9708.388857 | 6117.682478 |
| Switzerland | 7790.176941 | 9697.181637 | 6136.151292 |
| Andorra | 7814.885348 | 10083.1277 | 5943.342461 |
| Oman | 7833.774092 | 10427.13762 | 5753.448756 |
| Liberia | 7922.181935 | 9498.504334 | 6632.938605 |
| Canada | 7941.767825 | 10091.2538 | 6064.741829 |
| Libya | 7971.987525 | 9638.346501 | 6485.523246 |
| Jordan | 8009.333474 | 10254.7021 | 6143.653779 |
| Italy | 8119.670685 | 9764.594859 | 6790.848615 |
| Turkey | 8134.306402 | 10933.4451 | 6000.624573 |
| South Africa | 8172.986015 | 9014.122336 | 7411.265207 |
| France | 8290.028888 | 10436.34254 | 6552.160119 |
| Israel | 8291.444496 | 10161.72641 | 6679.748049 |
| Somalia | 8315.822022 | 9245.358639 | 8259.567778 |
| Bangladesh | 8358.879054 | 10266.91944 | 6765.339594 |
| Nepal | 8382.289025 | 10350.23994 | 6765.408331 |
| Rwanda | 8388.952215 | 10229.49437 | 6838.971686 |
| Yemen | 8425.255497 | 10165.82793 | 6883.524191 |
| United Kingdom | 8440.796995 | 10374.50265 | 6845.23406 |
| Australia | 8716.123627 | 11147.45644 | 6755.394709 |
| Eswatini | 8757.216848 | 11175.55954 | 6916.570463 |
| Monaco | 8801.84049 | 11900.11197 | 6418.951455 |
| Chile | 8851.047021 | 11500.02233 | 6614.472449 |
| Bahrain | 8908.895143 | 11752.43253 | 6641.058091 |
| San Marino | 8946.730623 | 11793.40545 | 6772.061801 |
| Democratic Republic of the Congo | 9030.643731 | 9510.995304 | 9101.062345 |
| Finland | 9212.657306 | 11442.44745 | 7357.213166 |
| Ireland | 9232.803821 | 11706.06634 | 7183.864551 |
| Morocco | 9413.656596 | 12037.29035 | 7301.921603 |
| Gabon | 9434.543892 | 11784.87307 | 7445.837459 |
| Afghanistan | 9473.146555 | 10090.13495 | 9485.26176 |
| Suriname | 9634.941209 | 12021.01248 | 7587.950753 |
| Central African Republic | 9760.405477 | 11789.91385 | 8239.319335 |
| Sweden | 9788.274143 | 11652.00535 | 8178.534605 |
| Congo | 9901.892959 | 12138.44158 | 8117.993229 |
| Equatorial Guinea | 9912.188915 | 12573.73902 | 7656.18174 |
| Iran (Islamic Republic of) | 9946.239661 | 11882.72241 | 8304.860864 |
| Guyana | 9955.916011 | 12573.61595 | 7754.948378 |
| Uganda | 9981.024473 | 12579.33534 | 7870.600159 |
| Gambia | 10074.22531 | 12657.24718 | 7886.580078 |
| Spain | 10080.6502 | 12690.96273 | 7996.060346 |
| Tunisia | 10137.07709 | 13278.47441 | 7607.118046 |
| Angola | 10169.56438 | 12843.98307 | 8061.274538 |
| Lebanon | 10271.28943 | 12724.85358 | 8274.648822 |
| Palestine | 10277.83053 | 13659.26986 | 7631.969559 |
| Portugal | 10290.15255 | 13086.58019 | 8111.601486 |
| United States of America | 10709.4616 | 12214.38964 | 9362.332616 |
| Greece | 10839.00409 | 13895.3862 | 8429.637633 |
| Lesotho | 11151.87705 | 14001.42235 | 8638.952229 |
| Greenland | 15623.41407 | 19563.9412 | 12323.07782 |

# Supplementary table 5. Age-scandalized DALYs rate (per100,000) of WCBA due to depressive disorders in 204 countries and territories in 2021 (ordered by value).

| **Location** | **Value** | **Upper** | **Lower** |
| --- | --- | --- | --- |
| Myanmar | 525.318299 | 738.3712276 | 348.8027547 |
| Colombia | 547.767182 | 790.297191 | 348.9539709 |
| Brunei Darussalam | 550.1964948 | 784.7004052 | 352.7409799 |
| China | 559.3983891 | 742.0195896 | 394.7805527 |
| Taiwan (Province of China) | 573.2466316 | 807.296623 | 371.9634625 |
| Poland | 597.7704871 | 804.1358236 | 422.761162 |
| Democratic People's Republic of Korea | 598.5382314 | 800.2596425 | 428.2820964 |
| Lao People's Democratic Republic | 611.163459 | 869.4808396 | 406.3336216 |
| American Samoa | 615.1971137 | 886.7311399 | 397.2950241 |
| Tonga | 641.2273052 | 907.4771236 | 433.8547315 |
| Samoa | 653.5509344 | 930.0122856 | 422.9397714 |
| Viet Nam | 657.2463725 | 949.9780425 | 422.3093548 |
| Indonesia | 658.5054785 | 897.7883416 | 460.0539918 |
| Singapore | 660.4048934 | 945.0691402 | 425.8984731 |
| Kiribati | 662.212901 | 919.4244704 | 448.9444256 |
| Micronesia (Federated States of) | 670.8088993 | 915.2321697 | 447.421022 |
| Marshall Islands | 673.2420689 | 928.8192406 | 463.7926229 |
| Northern Mariana Islands | 674.7221803 | 986.015627 | 430.8084046 |
| Seychelles | 682.3102067 | 951.6036709 | 455.2004399 |
| Thailand | 692.432951 | 950.798505 | 475.4107679 |
| Timor-Leste | 701.1935612 | 998.804214 | 456.7497984 |
| Vanuatu | 717.93452 | 1037.701752 | 455.2858141 |
| Republic of Korea | 720.1687564 | 1028.02308 | 475.6443028 |
| Papua New Guinea | 720.3214998 | 996.406289 | 480.3613247 |
| Solomon Islands | 730.1103095 | 997.1960052 | 514.2629681 |
| Fiji | 750.9120173 | 1083.761087 | 495.4902422 |
| Peru | 752.5916385 | 1107.504701 | 488.0897115 |
| Philippines | 769.4303166 | 1022.398185 | 554.7249251 |
| Cambodia | 776.4130977 | 1053.123794 | 547.3574199 |
| Tuvalu | 784.1591498 | 1126.936494 | 520.2218488 |
| Guam | 785.888233 | 1143.698687 | 507.6084084 |
| Niue | 788.1522807 | 1122.446732 | 522.1104012 |
| Nauru | 788.3670295 | 1156.100763 | 508.8084162 |
| Tokelau | 789.7679649 | 1167.852427 | 501.8807487 |
| Palau | 791.6869091 | 1178.594832 | 507.4041366 |
| Cook Islands | 793.4618682 | 1183.654404 | 504.2430892 |
| Sri Lanka | 806.4140641 | 1117.726763 | 564.9253213 |
| Puerto Rico | 808.7385263 | 1184.699319 | 488.3193957 |
| Malaysia | 816.0906448 | 1136.853313 | 552.9442504 |
| Japan | 823.3045022 | 1111.525671 | 578.8914736 |
| Hungary | 827.5437943 | 1151.827589 | 548.360814 |
| Maldives | 831.2692225 | 1173.631797 | 563.6003379 |
| Azerbaijan | 843.9839106 | 1186.185592 | 552.9807836 |
| Tajikistan | 847.8188716 | 1185.648648 | 578.6014619 |
| Zimbabwe | 856.2677654 | 1205.270985 | 580.7045572 |
| Romania | 856.955277 | 1189.611421 | 582.8981668 |
| Albania | 857.59898 | 1215.154945 | 586.3072861 |
| Uzbekistan | 864.5747142 | 1064.767282 | 716.7914441 |
| Serbia | 865.364627 | 1210.825994 | 581.4504612 |
| Turkmenistan | 867.8533099 | 1248.447582 | 561.8276946 |
| Nigeria | 871.3477631 | 1147.589069 | 624.9102949 |
| Croatia | 876.0525724 | 1212.430794 | 597.9856489 |
| Mauritania | 878.7249538 | 1239.160315 | 574.3609668 |
| Armenia | 883.7922175 | 1256.62616 | 583.4818163 |
| Mali | 908.4287357 | 1249.142185 | 627.8531921 |
| North Macedonia | 911.9802057 | 1191.757232 | 640.4306037 |
| Czechia | 915.748129 | 1306.654146 | 583.2197961 |
| Slovakia | 920.9100952 | 1346.586901 | 587.4678355 |
| Venezuela (Bolivarian Republic of) | 923.2442405 | 1310.522557 | 602.3818246 |
| Georgia | 936.6351252 | 1328.404379 | 600.7730118 |
| Bosnia and Herzegovina | 940.5670187 | 1357.117673 | 612.7842327 |
| C么te d'Ivoire | 964.1021391 | 1328.274956 | 636.2049278 |
| Bulgaria | 969.0231131 | 1393.088439 | 630.5452749 |
| Montenegro | 978.586004 | 1349.726222 | 659.1078133 |
| Bhutan | 979.32017 | 1446.334561 | 622.4332281 |
| Kazakhstan | 986.5256241 | 1406.607295 | 668.510192 |
| Panama | 989.0798931 | 1436.415467 | 646.5668488 |
| Republic of Moldova | 992.7580834 | 1361.114976 | 693.4750764 |
| Russian Federation | 997.988715 | 1313.063297 | 727.3983442 |
| Slovenia | 999.9654053 | 1468.124733 | 656.5141197 |
| Kyrgyzstan | 1007.318236 | 1383.942164 | 684.6531176 |
| Iceland | 1015.967905 | 1455.931401 | 643.9599241 |
| Antigua and Barbuda | 1021.556232 | 1506.055565 | 659.5851082 |
| Argentina | 1024.701869 | 1351.391438 | 714.9409705 |
| Sao Tome and Principe | 1027.462446 | 1468.324335 | 683.4278964 |
| Grenada | 1037.840333 | 1499.524906 | 667.9814461 |
| Dominica | 1039.415535 | 1515.452828 | 665.3708114 |
| Burkina Faso | 1039.724788 | 1507.446124 | 665.1794148 |
| Mongolia | 1039.754753 | 1524.744368 | 669.102149 |
| Belize | 1044.22745 | 1452.399939 | 698.3255215 |
| Senegal | 1048.464637 | 1471.792036 | 687.5305072 |
| United States Virgin Islands | 1050.475736 | 1548.788112 | 632.7534956 |
| Niger | 1051.012846 | 1475.079465 | 715.4164229 |
| India | 1055.259328 | 1354.854988 | 773.0138004 |
| Bahamas | 1057.732945 | 1504.016907 | 692.5175495 |
| Jamaica | 1066.159998 | 1567.071852 | 694.4203693 |
| Saint Vincent and the Grenadines | 1066.264072 | 1524.061428 | 685.4525247 |
| Bermuda | 1074.180481 | 1591.904573 | 659.66684 |
| Honduras | 1077.416957 | 1565.940598 | 673.9050551 |
| Barbados | 1081.018861 | 1539.079445 | 697.1473576 |
| Zambia | 1082.813296 | 1475.754025 | 738.710521 |
| Costa Rica | 1084.804745 | 1565.626307 | 700.3740619 |
| Malawi | 1101.285133 | 1574.12063 | 709.0381811 |
| Nicaragua | 1113.634036 | 1544.290698 | 779.5852296 |
| Togo | 1123.639783 | 1542.957014 | 767.3830701 |
| Saint Lucia | 1126.346598 | 1625.972361 | 741.3498085 |
| Comoros | 1129.223566 | 1531.511514 | 786.8291356 |
| Austria | 1134.933482 | 1633.760836 | 735.7127562 |
| Sierra Leone | 1135.997418 | 1647.492878 | 733.5548147 |
| Ghana | 1139.899398 | 1639.069861 | 773.1831034 |
| Pakistan | 1142.528795 | 1593.703881 | 767.1784979 |
| Benin | 1142.833465 | 1617.959716 | 769.7572574 |
| Ethiopia | 1148.500395 | 1486.635915 | 869.3441074 |
| Malta | 1156.057445 | 1637.532218 | 763.1611216 |
| Namibia | 1157.852031 | 1650.169634 | 777.8103557 |
| Luxembourg | 1168.228932 | 1624.95947 | 774.2078993 |
| Ecuador | 1173.728199 | 1643.870411 | 761.9347315 |
| Cyprus | 1180.532786 | 1663.897748 | 765.5216356 |
| Cameroon | 1190.774158 | 1588.571154 | 832.915145 |
| Estonia | 1191.721194 | 1724.946109 | 769.8237513 |
| Djibouti | 1196.735171 | 1632.780366 | 794.0253316 |
| Haiti | 1198.038639 | 1604.084127 | 816.8137002 |
| Cuba | 1199.672842 | 1711.194622 | 812.2062236 |
| Kenya | 1201.603897 | 1621.685079 | 831.7463577 |
| Guinea | 1203.696459 | 1711.878866 | 790.2328165 |
| El Salvador | 1208.364924 | 1711.330658 | 821.2151491 |
| United Republic of Tanzania | 1224.057772 | 1696.822811 | 809.5457019 |
| Latvia | 1226.565077 | 1773.1693 | 778.172914 |
| Guinea-Bissau | 1228.17143 | 1713.001772 | 828.0900824 |
| South Sudan | 1231.808732 | 1725.712196 | 837.1139298 |
| Dominican Republic | 1232.747705 | 1774.704795 | 793.6630834 |
| Kuwait | 1238.262279 | 1797.224423 | 815.3631905 |
| Eritrea | 1246.4518 | 1456.960018 | 1015.097634 |
| Madagascar | 1255.998818 | 1831.037653 | 778.5845073 |
| Guatemala | 1258.660252 | 1914.247154 | 778.0638125 |
| Ukraine | 1273.354086 | 1652.330132 | 955.9505058 |
| Germany | 1274.936509 | 1814.517429 | 836.5533926 |
| Burundi | 1287.92426 | 1797.451706 | 864.0868454 |
| New Zealand | 1290.620078 | 1813.03259 | 882.7823076 |
| Saint Kitts and Nevis | 1291.133159 | 1822.815768 | 859.9283952 |
| Mozambique | 1293.72094 | 1886.892081 | 845.4191917 |
| Mauritius | 1294.634178 | 1842.712891 | 859.9036833 |
| Iraq | 1299.101179 | 1635.175035 | 1079.645961 |
| United Arab Emirates | 1303.055221 | 1815.670194 | 910.8589514 |
| Denmark | 1309.135457 | 1862.160096 | 841.08373 |
| Bolivia (Plurinational State of) | 1318.075046 | 1858.671989 | 855.3882813 |
| Botswana | 1333.686042 | 1803.59912 | 890.2067282 |
| Uruguay | 1335.279598 | 1904.35657 | 877.9414789 |
| Cabo Verde | 1336.910007 | 1906.591201 | 890.3473226 |
| Chad | 1337.677601 | 1866.959057 | 914.7303781 |
| Paraguay | 1343.82715 | 1816.034949 | 941.5115853 |
| Belgium | 1348.664107 | 1903.602865 | 866.4980398 |
| Algeria | 1362.779073 | 1925.798391 | 895.4147304 |
| Egypt | 1369.797623 | 2012.414737 | 854.7944894 |
| Liberia | 1374.762577 | 1929.71428 | 947.7447902 |
| Sudan | 1379.347106 | 1931.834777 | 954.0001088 |
| Saudi Arabia | 1383.816363 | 1928.108795 | 942.5419578 |
| Syrian Arab Republic | 1385.835929 | 1955.347331 | 960.8551631 |
| Norway | 1392.999708 | 1884.937595 | 956.5315856 |
| Netherlands | 1398.574863 | 2030.780904 | 891.4002469 |
| Qatar | 1399.203079 | 2125.69979 | 870.3120797 |
| Switzerland | 1416.073873 | 1998.076498 | 924.2043528 |
| Brazil | 1420.960565 | 1820.783007 | 1053.640709 |
| Oman | 1421.743119 | 2148.23597 | 855.0127387 |
| Trinidad and Tobago | 1422.786715 | 2076.116292 | 945.8615096 |
| Canada | 1428.522106 | 2069.970607 | 913.31649 |
| South Africa | 1429.310525 | 1851.373179 | 1051.987175 |
| Belarus | 1431.065257 | 1944.101829 | 971.3749789 |
| Lithuania | 1433.122075 | 2048.225449 | 922.879154 |
| Andorra | 1437.563215 | 2091.145384 | 900.3794354 |
| Libya | 1441.790096 | 1995.408369 | 967.1502233 |
| Jordan | 1453.172374 | 2129.41081 | 917.6525956 |
| Somalia | 1453.955948 | 1848.30839 | 1161.837838 |
| Mexico | 1454.881852 | 2006.531674 | 993.4760107 |
| Rwanda | 1476.317369 | 2098.384745 | 975.6463705 |
| Turkey | 1495.079843 | 2275.98002 | 896.7087551 |
| Italy | 1504.313571 | 2062.383062 | 1034.029322 |
| Yemen | 1517.754779 | 2079.429657 | 1012.952278 |
| Israel | 1531.790382 | 2168.798365 | 1016.789779 |
| France | 1531.95128 | 2164.194052 | 1009.194293 |
| Bangladesh | 1532.239029 | 2157.787015 | 1000.657873 |
| Nepal | 1534.58862 | 2144.772807 | 1031.76446 |
| Eswatini | 1553.329011 | 2272.440578 | 982.6617067 |
| United Kingdom | 1556.879177 | 2209.569392 | 1041.058268 |
| Australia | 1616.634955 | 2328.750199 | 1045.966216 |
| Democratic Republic of the Congo | 1619.004235 | 1925.373592 | 1349.159746 |
| Bahrain | 1639.213673 | 2422.547181 | 1019.262183 |
| Monaco | 1639.592833 | 2502.659152 | 990.6784725 |
| San Marino | 1672.219121 | 2457.315902 | 1048.308255 |
| Chile | 1701.845595 | 2416.608419 | 1061.122004 |
| Gabon | 1704.851968 | 2447.656835 | 1104.690676 |
| Finland | 1708.731354 | 2402.444571 | 1145.783458 |
| Ireland | 1732.990972 | 2470.395575 | 1138.870216 |
| Afghanistan | 1738.494805 | 2089.194852 | 1453.080041 |
| Morocco | 1739.335331 | 2482.985246 | 1125.587865 |
| Central African Republic | 1769.987054 | 2376.480411 | 1263.285231 |
| Uganda | 1803.692621 | 2583.644257 | 1165.320384 |
| Equatorial Guinea | 1805.209515 | 2624.429904 | 1168.262748 |
| Congo | 1805.21391 | 2501.777438 | 1216.628996 |
| Sweden | 1833.594825 | 2505.093854 | 1265.643063 |
| Iran (Islamic Republic of) | 1834.460589 | 2511.512089 | 1266.741063 |
| Suriname | 1839.776072 | 2619.264717 | 1211.353254 |
| Gambia | 1841.433581 | 2658.639409 | 1170.353231 |
| Angola | 1864.142885 | 2686.552016 | 1177.28265 |
| Tunisia | 1891.132389 | 2776.173719 | 1203.396141 |
| Guyana | 1895.954216 | 2712.904603 | 1266.597174 |
| Spain | 1897.456023 | 2728.57783 | 1264.314862 |
| Lebanon | 1912.820184 | 2683.488792 | 1263.331991 |
| Palestine | 1923.435559 | 2874.141525 | 1188.479267 |
| Portugal | 1946.393833 | 2715.26484 | 1258.504962 |
| United States of America | 1982.287445 | 2651.549077 | 1430.336523 |
| Lesotho | 2030.26163 | 2808.123284 | 1325.631104 |
| Greece | 2062.842575 | 2993.309786 | 1345.485353 |
| Greenland | 3015.727097 | 4339.013683 | 1994.505264 |

# Supplementary table 6. AAPC of incidence due to depressive disorders among WCBA in 204 countries and territories from 1990 to 2021.

| **Location** | **AAPC** | **CI Low** | **CI High** | **P-Value** |
| --- | --- | --- | --- | --- |
| Afghanistan | 0.66 | 0.60 | 0.73 | <0.001 |
| Albania | 1.29 | 0.99 | 1.59 | <0.001 |
| Algeria | 0.42 | 0.17 | 0.66 | <0.001 |
| American Samoa | 0.38 | 0.25 | 0.50 | <0.001 |
| Andorra | 0.91 | 0.51 | 1.32 | <0.001 |
| Angola | 0.40 | 0.20 | 0.60 | <0.001 |
| Antigua and Barbuda | 0.90 | 0.86 | 0.93 | <0.001 |
| Argentina | 0.79 | 0.46 | 1.12 | <0.001 |
| Armenia | 1.35 | 1.18 | 1.53 | <0.001 |
| Australia | 0.53 | 0.42 | 0.64 | <0.001 |
| Austria | 0.13 | -0.07 | 0.34 | 0.192 |
| Azerbaijan | 1.17 | 0.98 | 1.35 | <0.001 |
| Bahamas | 1.12 | 0.90 | 1.34 | <0.001 |
| Bahrain | 0.31 | 0.22 | 0.40 | <0.001 |
| Bangladesh | 0.49 | 0.41 | 0.57 | <0.001 |
| Barbados | 1.05 | 0.99 | 1.11 | <0.001 |
| Belarus | 1.44 | 1.40 | 1.49 | <0.001 |
| Belgium | 1.27 | 0.64 | 1.90 | <0.001 |
| Belize | 1.08 | 0.87 | 1.29 | <0.001 |
| Benin | 0.37 | 0.31 | 0.43 | <0.001 |
| Bermuda | 0.38 | 0.33 | 0.44 | <0.001 |
| Bhutan | -0.21 | -0.34 | -0.08 | 0.002 |
| Bolivia (Plurinational State of) | 1.23 | 1.07 | 1.39 | <0.001 |
| Bosnia and Herzegovina | 0.31 | 0.20 | 0.41 | <0.001 |
| Botswana | 0.93 | 0.81 | 1.04 | <0.001 |
| Brazil | 0.75 | 0.38 | 1.11 | <0.001 |
| Brunei Darussalam | 0.65 | 0.56 | 0.73 | <0.001 |
| Bulgaria | 0.98 | 0.91 | 1.04 | <0.001 |
| Burkina Faso | 0.18 | -0.17 | 0.53 | 0.308 |
| Burundi | -0.09 | -0.15 | -0.02 | 0.007 |
| Cabo Verde | 1.07 | 0.87 | 1.28 | <0.001 |
| Cambodia | 0.00 | -0.09 | 0.09 | 0.972 |
| Cameroon | 0.33 | 0.30 | 0.36 | <0.001 |
| Canada | 1.21 | 1.06 | 1.36 | <0.001 |
| Central African Republic | 0.00 | -0.12 | 0.12 | 0.972 |
| Chad | 0.29 | 0.08 | 0.51 | 0.007 |
| Chile | 0.48 | 0.34 | 0.61 | <0.001 |
| China | -1.19 | -1.59 | -0.79 | <0.001 |
| Colombia | -0.05 | -0.42 | 0.32 | 0.790 |
| Comoros | 0.47 | 0.30 | 0.63 | <0.001 |
| Congo | 0.35 | 0.30 | 0.41 | <0.001 |
| Cook Islands | 0.44 | 0.36 | 0.51 | <0.001 |
| Costa Rica | 1.14 | 1.01 | 1.28 | <0.001 |
| Côte d'Ivoire | 0.09 | 0.01 | 0.17 | 0.027 |
| Croatia | 0.09 | 0.05 | 0.14 | <0.001 |
| Cuba | -0.82 | -0.91 | -0.73 | <0.001 |
| Cyprus | 0.70 | 0.62 | 0.78 | <0.001 |
| Czechia | 0.30 | 0.21 | 0.39 | <0.001 |
| Democratic People's Republic of Korea | -0.23 | -0.25 | -0.21 | <0.001 |
| Democratic Republic of the Congo | 0.21 | 0.00 | 0.41 | 0.048 |
| Denmark | -0.31 | -0.36 | -0.25 | <0.001 |
| Djibouti | 0.51 | 0.36 | 0.67 | <0.001 |
| Dominica | 0.95 | 0.89 | 1.00 | <0.001 |
| Dominican Republic | 0.75 | 0.32 | 1.19 | <0.001 |
| Ecuador | 1.25 | 1.15 | 1.35 | <0.001 |
| Egypt | 0.77 | 0.60 | 0.95 | <0.001 |
| El Salvador | 0.20 | -0.15 | 0.55 | 0.264 |
| Equatorial Guinea | 0.29 | 0.07 | 0.51 | 0.011 |
| Eritrea | 0.21 | 0.20 | 0.22 | <0.001 |
| Estonia | -0.16 | -0.23 | -0.09 | <0.001 |
| Eswatini | 1.60 | 1.54 | 1.66 | <0.001 |
| Ethiopia | 0.11 | 0.03 | 0.18 | 0.008 |
| Fiji | 0.60 | 0.57 | 0.63 | <0.001 |
| Finland | 0.43 | -0.06 | 0.92 | 0.087 |
| France | 0.30 | -0.09 | 0.69 | 0.132 |
| Gabon | 0.42 | 0.39 | 0.46 | <0.001 |
| Gambia | 0.23 | 0.18 | 0.28 | <0.001 |
| Georgia | 1.00 | 0.99 | 1.01 | <0.001 |
| Germany | 1.33 | 1.14 | 1.51 | <0.001 |
| Ghana | 0.35 | 0.19 | 0.51 | <0.001 |
| Greece | 0.88 | 0.18 | 1.58 | 0.014 |
| Greenland | 0.57 | 0.45 | 0.68 | <0.001 |
| Grenada | 0.86 | 0.81 | 0.90 | <0.001 |
| Guam | 0.77 | 0.66 | 0.88 | <0.001 |
| Guatemala | 0.79 | 0.42 | 1.16 | <0.001 |
| Guinea | 0.50 | 0.40 | 0.61 | <0.001 |
| Guinea-Bissau | 0.44 | 0.27 | 0.61 | <0.001 |
| Guyana | 1.37 | 1.31 | 1.42 | <0.001 |
| Haiti | 0.59 | 0.43 | 0.75 | <0.001 |
| Honduras | 1.26 | 1.16 | 1.37 | <0.001 |
| Hungary | -0.28 | -0.32 | -0.24 | <0.001 |
| Iceland | -0.03 | -0.19 | 0.12 | 0.682 |
| India | 0.07 | -0.08 | 0.21 | 0.370 |
| Indonesia | 0.94 | 0.87 | 1.00 | <0.001 |
| Iran (Islamic Republic of) | 0.49 | 0.28 | 0.71 | <0.001 |
| Iraq | -0.01 | -0.70 | 0.68 | 0.971 |
| Ireland | 1.34 | 1.16 | 1.53 | <0.001 |
| Israel | 0.53 | 0.43 | 0.63 | <0.001 |
| Italy | 0.95 | 0.50 | 1.40 | <0.001 |
| Jamaica | 0.95 | 0.91 | 0.99 | <0.001 |
| Japan | 1.12 | 0.87 | 1.37 | <0.001 |
| Jordan | 0.13 | 0.05 | 0.21 | 0.002 |
| Kazakhstan | 0.81 | 0.65 | 0.98 | <0.001 |
| Kenya | 0.17 | -0.09 | 0.43 | 0.211 |
| Kiribati | -0.01 | -0.06 | 0.05 | 0.818 |
| Kuwait | 0.04 | -0.29 | 0.38 | 0.809 |
| Kyrgyzstan | 0.83 | 0.70 | 0.95 | <0.001 |
| Lao People's Democratic Republic | -0.16 | -0.60 | 0.29 | 0.490 |
| Latvia | 0.45 | 0.22 | 0.68 | <0.001 |
| Lebanon | 1.58 | 1.55 | 1.62 | <0.001 |
| Lesotho | 0.89 | 0.84 | 0.95 | <0.001 |
| Liberia | 0.22 | 0.18 | 0.26 | <0.001 |
| Libya | 0.52 | 0.28 | 0.77 | <0.001 |
| Lithuania | 1.00 | 0.96 | 1.03 | <0.001 |
| Luxembourg | -0.12 | -0.37 | 0.12 | 0.324 |
| Madagascar | 0.43 | 0.31 | 0.56 | <0.001 |
| Malawi | 0.28 | 0.25 | 0.32 | <0.001 |
| Malaysia | 0.54 | -0.10 | 1.18 | 0.098 |
| Maldives | -0.55 | -0.61 | -0.48 | <0.001 |
| Mali | 0.15 | 0.08 | 0.23 | <0.001 |
| Malta | 0.77 | 0.52 | 1.02 | <0.001 |
| Marshall Islands | 0.26 | 0.19 | 0.32 | <0.001 |
| Mauritania | 0.23 | 0.13 | 0.33 | <0.001 |
| Mauritius | 0.29 | 0.21 | 0.36 | <0.001 |
| Mexico | 2.30 | 2.14 | 2.46 | <0.001 |
| Micronesia (Federated States of) | 0.10 | 0.03 | 0.16 | 0.003 |
| Monaco | 0.93 | 0.90 | 0.97 | <0.001 |
| Mongolia | -0.07 | -0.20 | 0.06 | 0.302 |
| Montenegro | 1.15 | 1.01 | 1.29 | <0.001 |
| Morocco | 0.60 | 0.23 | 0.96 | 0.001 |
| Mozambique | 0.66 | 0.64 | 0.69 | <0.001 |
| Myanmar | 1.06 | 0.78 | 1.34 | <0.001 |
| Namibia | 1.01 | 0.60 | 1.43 | <0.001 |
| Nauru | 0.45 | 0.36 | 0.53 | <0.001 |
| Nepal | 1.33 | 1.26 | 1.41 | <0.001 |
| Netherlands | 0.79 | 0.61 | 0.97 | <0.001 |
| New Zealand | 0.36 | 0.21 | 0.51 | <0.001 |
| Nicaragua | 0.88 | 0.74 | 1.01 | <0.001 |
| Niger | -0.07 | -0.12 | -0.02 | 0.009 |
| Nigeria | -0.48 | -0.77 | -0.20 | <0.001 |
| Niue | 0.44 | 0.35 | 0.52 | <0.001 |
| North Macedonia | 1.18 | 1.06 | 1.29 | <0.001 |
| Northern Mariana Islands | 0.92 | 0.86 | 0.98 | <0.001 |
| Norway | 0.82 | 0.69 | 0.94 | <0.001 |
| Oman | 0.78 | 0.71 | 0.85 | <0.001 |
| Pakistan | 0.49 | 0.13 | 0.84 | 0.007 |
| Palau | 0.44 | 0.36 | 0.52 | <0.001 |
| Palestine | 0.59 | 0.45 | 0.73 | <0.001 |
| Panama | 1.00 | 0.56 | 1.45 | <0.001 |
| Papua New Guinea | -0.05 | -0.13 | 0.03 | 0.237 |
| Paraguay | 1.23 | 1.15 | 1.31 | <0.001 |
| Peru | 0.98 | 0.30 | 1.67 | 0.005 |
| Philippines | 0.57 | 0.48 | 0.67 | <0.001 |
| Poland | 0.81 | 0.71 | 0.90 | <0.001 |
| Portugal | 0.76 | -0.19 | 1.71 | 0.117 |
| Puerto Rico | 0.79 | 0.45 | 1.12 | <0.001 |
| Qatar | 0.10 | -0.15 | 0.35 | 0.442 |
| Republic of Korea | 0.76 | 0.61 | 0.92 | <0.001 |
| Republic of Moldova | 0.40 | 0.29 | 0.52 | <0.001 |
| Romania | 1.05 | 0.94 | 1.17 | <0.001 |
| Russian Federation | 0.98 | 0.88 | 1.08 | <0.001 |
| Rwanda | -0.05 | -0.14 | 0.05 | 0.320 |
| Saint Kitts and Nevis | 0.81 | 0.74 | 0.89 | <0.001 |
| Saint Lucia | 1.10 | 1.06 | 1.14 | <0.001 |
| Saint Vincent and the Grenadines | 0.95 | 0.91 | 1.00 | <0.001 |
| Samoa | 0.15 | 0.07 | 0.22 | <0.001 |
| San Marino | 1.13 | 1.08 | 1.18 | <0.001 |
| Sao Tome and Principe | 0.35 | 0.21 | 0.49 | <0.001 |
| Saudi Arabia | 0.73 | 0.36 | 1.10 | <0.001 |
| Senegal | 0.70 | 0.52 | 0.89 | <0.001 |
| Serbia | 0.40 | 0.31 | 0.49 | <0.001 |
| Seychelles | 0.88 | 0.85 | 0.91 | <0.001 |
| Sierra Leone | 0.38 | 0.31 | 0.45 | <0.001 |
| Singapore | -1.34 | -1.48 | -1.19 | <0.001 |
| Slovakia | 0.80 | 0.57 | 1.03 | <0.001 |
| Slovenia | -0.02 | -0.16 | 0.12 | 0.799 |
| Solomon Islands | 0.15 | 0.07 | 0.23 | <0.001 |
| Somalia | 0.74 | 0.69 | 0.78 | <0.001 |
| South Africa | 1.06 | 0.73 | 1.39 | <0.001 |
| South Sudan | 0.36 | 0.19 | 0.53 | <0.001 |
| Spain | 1.62 | 1.49 | 1.76 | <0.001 |
| Sri Lanka | -0.23 | -0.31 | -0.15 | <0.001 |
| Sudan | 0.29 | -0.01 | 0.58 | 0.058 |
| Suriname | 1.29 | 1.25 | 1.33 | <0.001 |
| Sweden | 0.63 | 0.23 | 1.03 | 0.002 |
| Switzerland | 0.00 | -0.13 | 0.14 | 0.967 |
| Syrian Arab Republic | 0.69 | 0.59 | 0.78 | <0.001 |
| Taiwan (Province of China) | 0.65 | 0.60 | 0.70 | <0.001 |
| Tajikistan | 0.64 | 0.53 | 0.74 | <0.001 |
| Thailand | 0.23 | 0.17 | 0.29 | <0.001 |
| Timor-Leste | -0.07 | -0.13 | 0.00 | 0.037 |
| Togo | 0.23 | 0.07 | 0.38 | 0.003 |
| Tokelau | 0.43 | 0.36 | 0.51 | <0.001 |
| Tonga | 0.24 | 0.17 | 0.30 | <0.001 |
| Trinidad and Tobago | 0.76 | 0.66 | 0.87 | <0.001 |
| Tunisia | 0.96 | 0.88 | 1.04 | <0.001 |
| Turkey | 0.68 | 0.42 | 0.94 | <0.001 |
| Turkmenistan | 0.55 | 0.48 | 0.61 | <0.001 |
| Tuvalu | 0.42 | 0.36 | 0.48 | <0.001 |
| Uganda | 0.32 | 0.25 | 0.40 | <0.001 |
| Ukraine | 0.66 | 0.62 | 0.70 | <0.001 |
| United Arab Emirates | 0.37 | 0.33 | 0.41 | <0.001 |
| United Kingdom | 0.64 | 0.20 | 1.08 | 0.004 |
| United Republic of Tanzania | 0.31 | 0.27 | 0.34 | <0.001 |
| United States of America | 2.05 | 1.52 | 2.58 | <0.001 |
| United States Virgin Islands | 0.87 | 0.71 | 1.03 | <0.001 |
| Uruguay | 1.33 | 1.23 | 1.42 | <0.001 |
| Uzbekistan | 0.41 | 0.30 | 0.52 | <0.001 |
| Vanuatu | 0.14 | 0.06 | 0.23 | 0.001 |
| Venezuela (Bolivarian Republic of) | 0.39 | 0.23 | 0.55 | <0.001 |
| Viet Nam | 0.20 | -0.03 | 0.44 | 0.087 |
| Yemen | -0.15 | -0.21 | -0.08 | <0.001 |
| Zambia | 0.46 | 0.40 | 0.52 | <0.001 |
| Zimbabwe | 0.66 | 0.47 | 0.86 | <0.001 |
