# Supplementary material for: Global, regional, and national burden and temporal trends of depressive disorders in women of child-bearing age, 1990 to 2021: A worldwide analysis
Source: Medicine (Baltimore). 2025 Oct 24;104(43):e45215. doi: 10.1097/MD.0000000000045215 (PMC12558287; doi:10.1097/MD.0000000000045215)

Supplementary Figure 1. Global trends of age-standardized rate of DD burden changes from 1990 to 2021 according to five SDI regions.

(A) Age-standardized DALYs rate, (B) Age−standardized prevalence rate (per 100000) and (C) Age-standardized incidence rate (per 100000). DALYs, disability-adjusted life years; SDI, Socio-demographic Index.


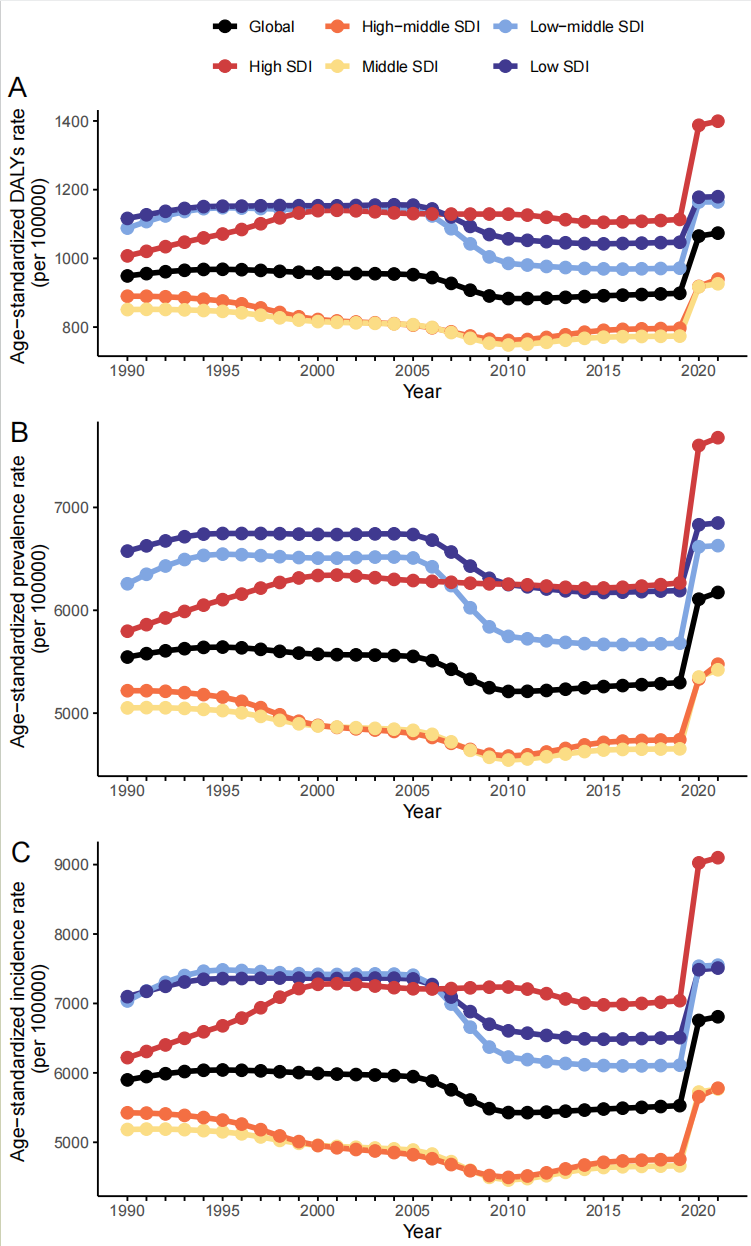


# Supplementary Figure 2. The spatial distribution of the age-standardized rate of prevalence due to depressive disorders among WCBA in 204 countries and territories.

(A) the rate of prevalence due to depressive disorders among WCBA in 2021, (B) the AAPC of the rate of prevalence due to depressive disorders among WCBA from 1990 to 2021. AAPC, average annual percentage change.


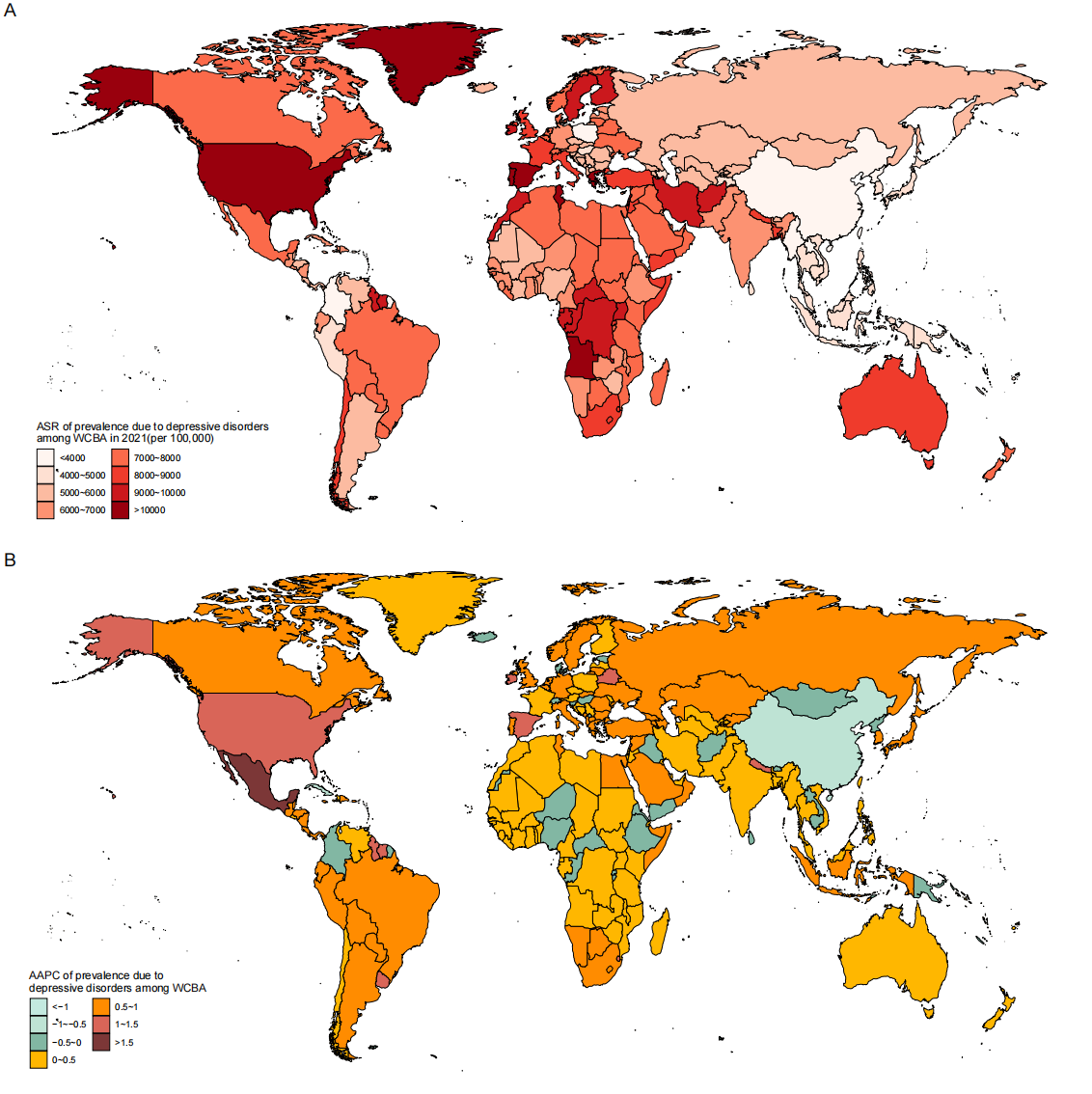


# Supplementary Figure 3. The spatial distribution of the rate of DALYs due to depressive disorders among WCBA in 204 countries and territories.

(A) the rate of DALYs due to depressive disorders among WCBA in 2021, (B) the AAPC of the rate of DALYs due to depressive disorders among WCBA from 1990 to 2021. DALYs, disability-adjusted life years; AAPC, average annual percentage change.


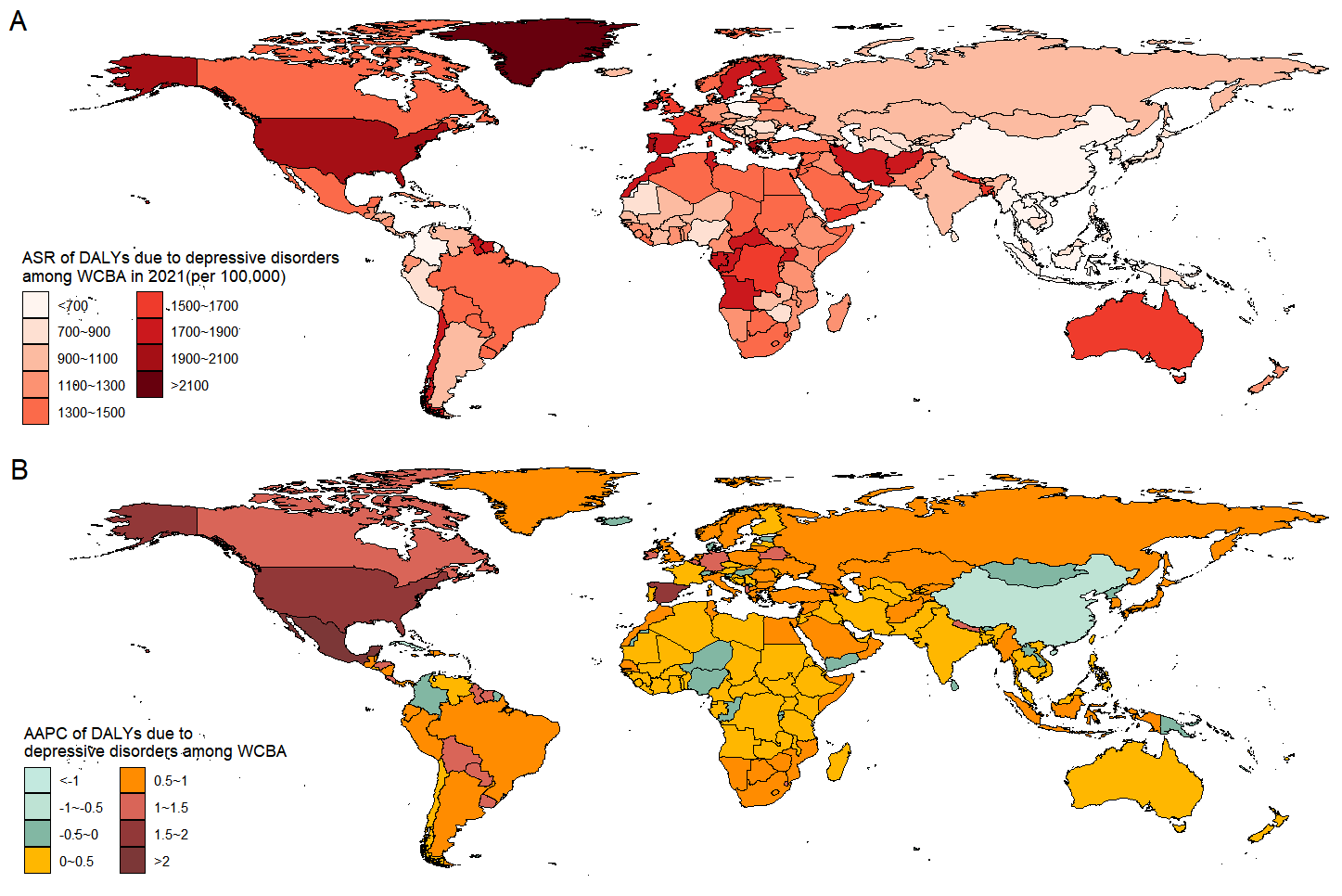


# Supplementary Figure 4. Trends in age-standardized rates per 100 000 among WCBA from 2021 to 2030 predicted by Bayesian age–period–cohort (BAPC) models. (A) DALYs rate; (B) Prevalence rate ;(C) Incidence rate.


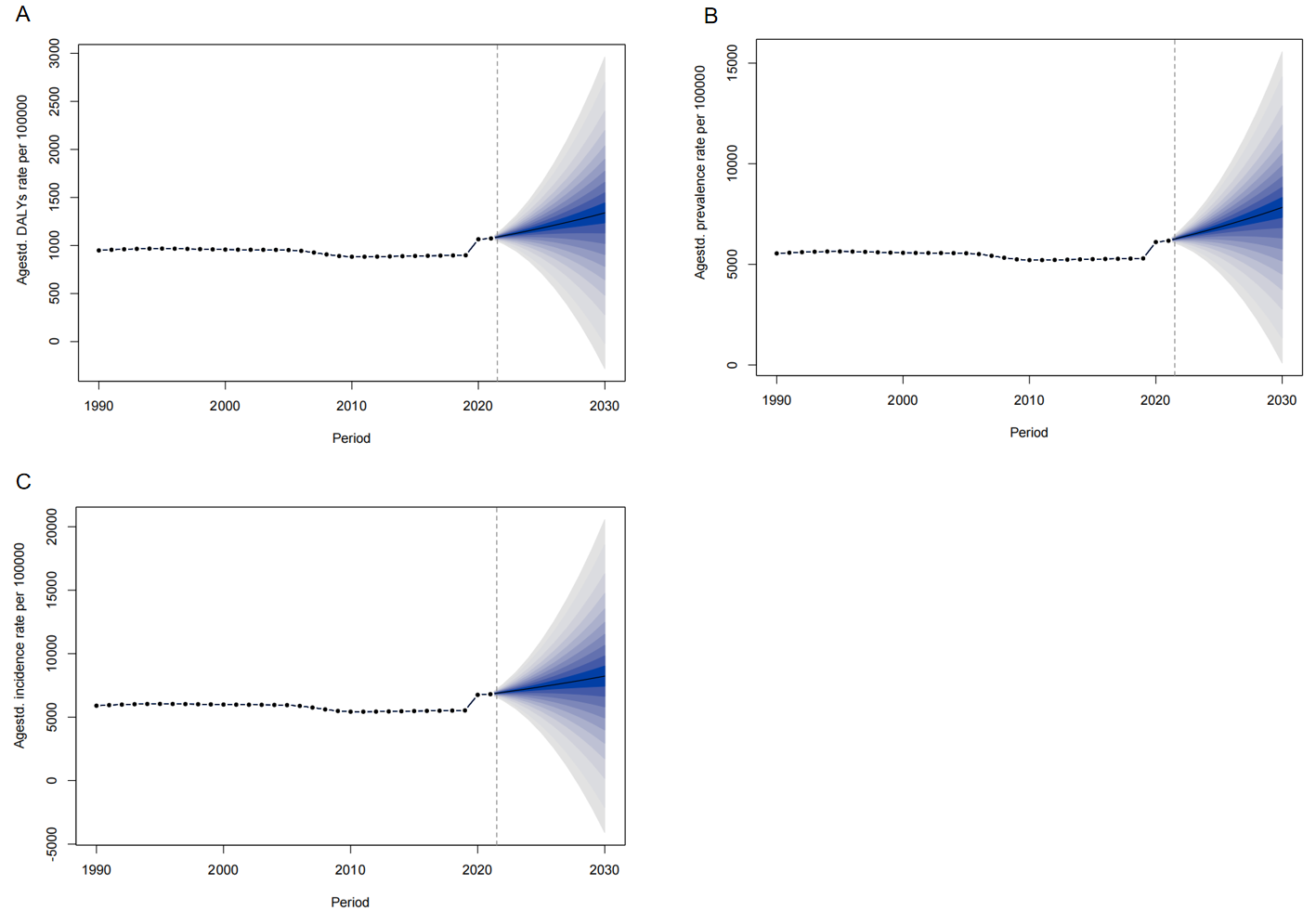

Supplement: Supplementary file 2 [file medi-104-e45215-s002.docx]
